# Epsin3 promotes non-small cell lung cancer progression via modulating EGFR stability

**DOI:** 10.1186/s13578-025-01358-1

**Published:** 2025-02-05

**Authors:** Huiling Su, Jie Shen, Chenzi Gao, Yue Zhao, Wanyu Deng, Bo Qin, Xin Zhang, Juan Lai, Qian Wang, Jie Dou, Min Guo

**Affiliations:** 1 State Key Laboratory of Natural Medicines, School of Life Science & Technology, Pharmaceutical University, 210009 Nanjing, China; 2 Key Laboratory of Human Functional Genomics of Jiangsu Province, Department of Biochemistry and Molecular Biology, Nanjing Medical University, 210029 Nanjing, China; 3 College of Life Science, Shangrao Normal University, 334001 Shangrao, China; 4 Shaoxing Women and Children’s Hospital, 312000 Shaoxing, China; 5 GeneMind Biosciences Company Limited, 518001 Shenzhen, China

**Keywords:** Non-small cell lung cancer, Epsin3, EGFR, TKI resistance

## Abstract

**Background:**

The abnormal expression and overactivation of the epidermal growth factor receptor (EGFR), a typical cancer marker for non-small cell lung cancer (NSCLC), are closely related to the tumorigenesis and progression of NSCLC. However, the endocytosis mechanism of EGFR in lung cancer is not yet known. Epsin3 (EPN3), a member of the endocytic adaptor protein family, is essential for the endocytosis of multiple receptors. In this study, we aimed to investigate the role of EPN3 in modulating EGFR function, its effects on NSCLC progression, and its potential involvement in tyrosine kinase inhibitor (TKI) resistance, which remains a significant hurdle in NSCLC treatment.

**Results:**

Our findings revealed that the expression of EPN3 is significantly up-regulated in NSCLC patients. Elevated EPN3 expression was proportional to shorter overall survival in patients with NSCLC. Functional analyses revealed that EPN3 directly interacts with EGFR, enhancing its recycling to the plasma membrane and preventing its degradation via the lysosomal pathway. This stabilization of EGFR led to sustained downstream signalling, promoting NSCLC cell proliferation and migration. Notably, mutations in the EGFR tyrosine kinase domain, which typically confer resistance to TKIs, did not alter the regulatory effect of EPN3.

**Conclusions:**

EPN3 enhances EGFR signalling by promoting its recycling and stability, contributing to NSCLC progression and TKI resistance. Targeting EPN3 could offer a novel therapeutic strategy to overcome drug resistance in EGFR-driven NSCLC.

**Supplementary Information:**

The online version contains supplementary material available at 10.1186/s13578-025-01358-1.

## Introduction

Non-small cell lung cancer (NSCLC) is characterized by a high incidence of rapid cancer cell proliferation and metastasis [1–3]. Conventional therapies such as surgery can improve the condition of only some early-stage patients. Owing to the current inadequate diagnostic conditions, the 5-year survival rate of most patients with stage IIIA NSCLC is only 10 − 30% [4]. Additionally, the recurrence and metastasis of cancer cells also result in a poor prognosis for advanced patients.

Epidermal growth factor receptor (EGFR) is recognized as an effective target for NSCLC treatment [5, 6]. Numerous studies have shown that mutations and abnormal activation of EGFR are closely related to tumour progression and treatment resistance in various epithelial malignancies [7–9]. Currently, two main categories of targeted therapy drugs are used for advanced, metastatic, or recurrent NSCLC: monoclonal antibodies (e.g., cetuximab and panitumumab) and small molecule kinase inhibitors (e.g., gefitinib and erlotinib), which bind to the extracellular region of EGFR [10, 11]. Small molecule kinase inhibitors, known as EGFR-tyrosine kinase inhibitors (EGFR-TKIs), specifically target the tyrosine kinase activity of EGFR, effectively blocking its intracellular autophosphorylation process and downstream activity signals [12]. EGFR-TKIs have demonstrated significant clinical efficacy in NSCLC patients. However, due to tumour heterogeneity [13], secondary gene mutations [14], aberrant activation of alternative signalling pathways [15], and other secondary drug resistance mechanisms have appeared that greatly reduce the cure rate of NSCLC. Most NSCLC patients develop drug resistance 9–14 months after treatment with first- or second-generation EGFR-TKIs. The most common resistance mechanism is the EGFR T790M mutation, which spatially hinders the interaction of the TKIs with the ATP-binding site of EGFR, preventing its ability to inhibit its activity. To overcome T790M-mediated resistance, third-generation EGFR-TKIs represented by osimertinib emerged as the times require, which can selectively inhibit T790M mutated EGFR [16]. Unfortunately, with the emergence of new EGFR mutations and resistance mechanisms, resistance to third-generation EGFR-TKIs has also occurred [17]. Therefore, exploring abnormally expressed genes and potential molecular mechanisms in NSCLC is pivotal to gain insights into the pathogenesis of NSCLC and overcome resistance.

Furthermore, studies have revealed that the spatial distribution and stability of EGFR are crucial factors in regulating lung cancer progression. Dysregulation of EGFR degradation further accelerates tumorigenesis and advancement, particularly in mutant EGFR-driven lung adenocarcinoma (LUAD) [18–20]. The complete activation and termination of EGFR signalling rely on ligand-induced endocytosis and intracellular trafficking [21]. Elevated EGFR levels can increase receptor signalling, promote malignant transformation, provide cancer cells with survival benefits, and foster drug resistance [19, 22]. However, the molecular mechanisms governing abnormal EGFR trafficking are poorly understood. Hence, it is crucial to elucidate the intricate molecular mechanisms underlying EGFR internalization and discover novel targets regulating EGFR transport and degradation. These findings have significant implications for treating NSCLC patients, especially those resistant to EGFR-TKIs.

The Epsin protein family is a class of highly evolutionarily conserved endocytic adapter proteins that are vital for fundamental cellular processes in both embryos and adults [23]. These proteins facilitate clathrin-mediated endocytosis and actively participate in regulating various signalling receptors, including receptor tyrosine kinases, Notch receptors, and Wnt receptors. Dysregulation of these receptor activation processes is closely associated with tumour occurrence [24–26]. In mammals, Epsins include Epsin1 (EPN1), Epsin2 (EPN2), and Epsin3 (EPN3) [27]. Previous studies have shown that both EPN1 and EPN2 are upregulated in various cancer types, which is correlated with tumour cell proliferation, migration, and invasion [28–30]. EPN3 has been demonstrated to influence tumour progression in breast cancer, oral squamous cell carcinoma, and glioblastoma [31–33]. Recent studies have also shown that EPN3 plays an oncogenic role in NSCLC by activating the JAK1/2-STAT3 pathway and that inhibiting EPN3 can reduce the metastasis and invasion ability of LUAD cells by inhibiting the EMT process [34, 35]. However, existing research has focused mainly on the impact of EPN3 on the proliferation, metastasis and invasion ability of NSCLC, and the mechanisms involved are also very simple. Notably, the relationship between EPN3 and EGFR has yet to be elucidated, and its influence on TKI resistance has not been thoroughly investigated.

In this study, we present novel evidence demonstrating that EPN3 affects tumour progression through interacting with EGFR and inhibiting the lysosomal degradation pathway of EGFR. This mechanism differs significantly from those previously reported, suggesting a broader and more complex role for EPN3 in NSCLC than currently appreciated. Our findings provide new insights that could challenge the existing paradigms of EPN3 function and highlight potential therapeutic targets for intervention.

## Materials and methods

### Chemicals and reagents

The CCK-8 cell counting kit (CCK-8), TRIzol reagent, and HiScript III 1st strand cDNA synthesis kit (+ gDNA wiper) were obtained from Nanjing Vazyme Biotechnology Co., Ltd. (Nanjing, China). Dulbecco’s modified Eagle’s medium (DMEM) and foetal bovine serum (FBS) were obtained from Gibco (Carlsbad, CA, USA). Hoechst 33342 staining solution, propidium iodide (PI) staining solution, a cell cycle analysis kit, penicillin‒streptomycin solution, and puromycin were purchased from Beyotime Institute of Biotechnology (Shanghai, China). T4 ligase was purchased from Takara Biomedical Technology Co., Ltd. (Beijing, China). Chloroquine (CQ), MG132, and epidermal growth factor (EGF) were purchased from MedChemExpress (Shanghai, China). All other reagents used were analytically pure.

### Cell lines, animals and clinical tissue samples

The human LUAD cell line A549 (RRID: CVCL_0023), NCI-H1975 (RRID: CVCL_1511), NCI-H1299 (RRID: CVCL_0060), human lung squamous cell carcinoma (LUSC) cell line NCI-H1703 (RRID: CVCL_1490) and human embryonic kidney cell line HEK-293T (RRID: CVCL_0063) were purchased from the National Collection of Authenticated Cell Cultures (Shanghai, China). The normal human bronchial epithelial cell line 16HBE (RRID: CVCL_0112) was purchased from the American Type Culture Collection (Manassas, VA, USA). These cell lines were cultured in DMEM containing 10% FBS, 100 U/mL penicillin and 100 µg/mL streptomycin and incubated in an atmosphere at 37 °C with 5% carbon dioxide. The cell lines were STR profiled and regularly tested for mycoplasma contamination, and the results were consistently negative.

SPF male BALB/c nude mice (18 ± 2 g) were purchased from Vital River Laboratory Animal Technology Co., Ltd. (Beijing, China), and had free access to food and water under controlled conditions of temperature (22–25 °C), humidity (55–70%) and light/dark cycle (12 h/12 h) in accordance with the Institutional Animal Care and Use Committee of China Pharmaceutical University.

All clinical tissue samples used in this study were obtained from Jiangsu Provincial People’s Hospital affiliated with Nanjing Medical University. With informed consent, 31 original patients who underwent lung cancer resection at Jiangsu Provincial People’s Hospital (Nanjing, China) from 2016 to 2019 were selected. The diagnosis was confirmed by histopathological examination, and no local or systemic treatment was given before the operation. All the tissue samples were stored in liquid nitrogen. All experiments were performed in accordance with the ethical standards of Nanjing Medical University and the world-recognized ethical standards, approved by the Ethics Committee of Nanjing Medical University.

### Knockdown plasmid construction

The lentiviral packaging plasmids psPAX2 and pMD2.G were donated by Nanjing Medical University. The lentiviral vector pLKO.1 expressing negative control shRNA (shNC) or shEPN3 was constructed. shRNA targeting human EPN3 was synthesized by TsingkeBiotech Co., Ltd. (Beijing, China). The shRNA sequences are listed in Table 1.

**Table 1:** Primer sequences

Primer	Sequence
shEPN3#1-F	5’-CCGG-GTGTACAAGGCTCTAACATTGCTCGAGCAATGTTAGAGCCTTGTACACTTTTTG-3’
shEPN3#1-R	5’-AATTCAAAAA-GTGTACAAGGCTCTAACATTG-CTCGAGCAATGTTAGAGCCTTGTACAC-3’
shEPN3#2-F	5’-CCGGAGTGGCCTTCACCGAAGTCATCTCGAGATGACTTCGGTGAAGGCCACTTTTTTG-3’
shEPN3#2-R	5’-AATTCAAAAAAGTGGCCTTCACCGAAGTCATCTCGAG-ATGACTTCGGTGAAGGCCACT-3’

### Overexpression plasmid construction

The truncations of EPN3-FL, M1 (amino acids 1-255), M2 (amino acids 1-144), M3 (amino acids 1-144 and amino acids 256–632), and anti-M2 (amino acids 144–632) were inserted into the pXJ40-HA vector. EGFR, the extracellular domain (ECD) (amino acids 1-644), the intracellular domain (ICD) (amino acids 644–1186), the juxtamembrane (JM) region (amino acids 644–687), the tyrosine kinase (TK) region (amino acids 688–955) and the C-terminal (CT) region (amino acids 956–1186) were inserted into the pEGFP-C3 vector by standard subcloning. The sequences of primers used for PCR reaction are listed in Table 2.

**Table 2:** Primer sequences

Primer	Sequence
EPN3-F	5’-CCCAAGCTTATGACGACCTCCGCACTCCG-3’
EPN3-R	5’-GGGGTACCTCAGAGGAAGGGGTTGGTGCCGGTCTGC-3’
M1-F	5’-CCCAAGCTTATGACGACCTCCGCAC-3’
M1-R	5’-GGGGTACCTCACTCCTTCTCGTGCTCCTGC-3’
M2-F	5’-CCCAAGCTTATGACGACCTCCGCAC-3’
M2-R	5’-GGGGTACCTCATCGCTCCTGCCGCAGC-3’
M3-F1	5’-CCCAAGCTTATGACGACCTCCGCAC-3’
M3-R1	5’-CAGgacctcacctcTCGCTCCTGCCGCAGCCG-3’
M3-F2	5’-GTGAGGTCCTGGCAGGGTG-3’
M3-R2	5’-GGGGTACCTCAGAGGAAGGGGTTGGTGC-3’
Anti-M2-F	5’-CCCAAGCTTATGCGAACCCACGCCCTCAAGACC-3’
Anti-M2-R	5’-GGGGTACCTCAGAGGAAGGGGTTG-3’
EGFR-F	5’-CCCAAGCTTATGCGACCCTCCGGGACGG-3’
EGFR-R	5’-GGGGTACCTCATGCTCCAATAAATTCACTGCTTTGTG-3’
ECD-F	5’-CCCAAGCTTATGCGACCCTCCGGGACGG-3’
ECD-R	5’-GGGGTACCTCACATGAAGAGGCCGATCCCC-3’
ICD-F	5’-CCCAAGCTTATGCGAAGGCGCCACATCGTT-3’
ICD-R	5’-GGGGTACCTCATGCTCCAATAAATTCACTGCTTTGTG-3’
JM-F	5’-CCCAAGCTTATGCGAAGGCGCCACATCGTT-3’
JM-R	5’-GGGGTACCTCATTCAGTTTCCTTCAAGATCCTCAA-3’
TK-F	5’-CCCAAGCTTATGTTCAAAAAGATCAAAGTGCTGGG-3’
TK-R	5’-GGGGTACCTCAAAGGTAGCGCTGGGGGTCT-3’
CT-F	5’-CCCAAGCTTATGGTCATTCAGGGGGATGAAAGAA-3’
CT-R	5’-GGGGTACCTCATGCTCCAATAAATTCACTGCTTTGTG-3’

### Generation of cell lines

To establish cell lines stably expressing EPN3-shRNA#1/2 or shNC, cells were transfected with EPN3-shRNA#1/2- or shNC-expressing plasmids. Stable transfectants were selected in medium containing puromycin. To establish HA-EPN3-expressing H1299 cells, H1299 cells were transfected with Lipofectamine^®^ 2000 transfection reagent (Thermo Fisher Scientific, Waltham, USA) according to the manufacturer’s instructions. To establish cells expressing wild-type (WT)-EGFR, Del19/T790M/C797S-EGFR, HA-EPN3/M1/M2/M3/antiM2 or green fluorescent protein (GFP)-EGFR/ECD/ICD/JM/TK/CT, 293T cells were transfected with Lipofectamine^®^ 2000 transfection reagent according to the manufacturer’s instructions.

### Quantitative real‑time PCR analysis

Total RNA extracted from mouse lung homogenate or human lung cancer cells by TRIzol was used for cDNA synthesis with the HiScript III 1st strand cDNA synthesis kit (+ gDNA wiper) following the manuscript’s instructions. The expression levels of the following genes were analysed with quantitative real-time polymerase chain reaction (qPCR) assays using the QuantStudio™3 Real-Time PCR system. The primer sequences are respectively listed in Table 3. The mRNA levels were calculated via the 2^−ΔΔCt^ method.

**Table 3:** Primer sequences

Gene	Sequence
GAPDH-F	5’-AAATCAAGTGGGGCGATGCTG-3’
GAPDH-R	5’-GCAGAGATGATGACCCTTTTG-3’
EPN3-F	5’-CTTGGCTGACATCTTCGTACCT-3’
EPN3-R	5’-TGTGTTCGGCCTAAAACCTG-3’
EGFR-F	5’-AGGCACGAGTAACAAGCTCAC-3’
EGFR-R	5’-ATGAGGACATAACCAGCCACC-3’

### Western blotting

The cells were seeded in a 6-well plate at 2 × 10^5^ cells per well and cultured at 37 °C with 5% CO_2_. Then, the cells were lysed with RIPA buffer (Epizyme Biomedical Technology Co., Ltd., Shanghai, China) containing 1% PMSF (Sigma, St. Louis, MO, USA) and phosphatase inhibitor cocktail (Beyotime Institute of Biotechnology). The concentration of protein lysate was determined by a BCA assay (Vazyme Biotech Co., Ltd.). Equivalent amounts of protein were mixed with 5×SDS-PAGE sample loading buffer (Beyotime Institute of Biotechnology). The protein levels were subsequently analysed using Western blotting as previously described [36], and incubated with anti-EPN3 (PA5-101583, Thermo Fisher Scientific, 1:1,000), anti-PARP1 (ab191217, Abcam, Cambridge, UK, 1:1,000), anti-cleaved PARP1 (ab110315, Abcam, 1:1,000), anti-caspase-3 (9662 S, Cell Signaling Technology, MA, USA, 1:1,000), anti-cleaved caspase-3 (9664 S, Cell Signaling Technology,1:1,000), anti-β-tubulin (2146 S, Cell Signaling Technology, 1:1,000), anti-Bax (41162 S, Cell Signaling Technology, 1:1,000), anti-Bcl-2 (15071 S, Cell Signaling Technology, 1:1,000), anti-E-cadherin (3195 S, Cell Signaling Technology, 1:1,000), anti-N-cadherin (13116 S, Cell Signaling Technology, 1:1,000), anti-Vimentin (5741 S, Cell Signaling Technology, 1:1,000), anti-EGFR (4267 S, Cell Signaling Technology, 1:1,000), anti-mTOR (2983 S, Cell Signaling Technology, 1:1,000), anti-p-mTOR (5536 S, Cell Signaling Technology, 1:1,000), anti-AKT (4685 S, Cell Signaling Technology, 1:1,000), anti-p-AKT (4060 S, Cell Signaling Technology, 1:2,000), anti-PI3K (4255 S, Cell Signaling Technology, 1:1,000), anti-HA (5017 S, Cell Signaling Technology, 1:1,000), and anti-GFP (2956 S, Cell Signaling Technology, 1:1,000), and probed with secondary antibody (ABclonal, Wuhan, China) conjugated with horseradish peroxidase at 37 °C for 2 h. Finally, the bands of protein on the membrane were visualized by electrochemiluminescence to analyse the protein levels and the band intensities of proteins were quantified using ImageJ software.

### Immunofluorescence

Immunofluorescence analysis was performed as previously described [36]. Cells seeded on coverslips were briefly washed with PBS, fixed with 4% paraformaldehyde for 15 min, permeabilized with 0.5% Triton X-100 for 15 min, blocked with 3% BSA for 30 min at 37 °C and stained with primary antibodies followed by corresponding secondary antibodies. Nuclei were counterstained with DAPI. Confocal images were taken with a confocal microscope (Zeiss, Jena, Germany).

### CCK-8 and colony formation assays

The cells were seeded on a 96-well plate with 1 × 10^4^ cells per well, and cultured at 37 °C with 5% CO_2_. The cell proliferation rate was evaluated by a CCK-8 assay according to the manufacturer’s instructions.

Colony formation assays were performed on 6-well plates. The cells were added to a 6-well plate at a density of 100 cells/well. After 14 days, the cells were washed twice with PBS and fixed with 4% paraformaldehyde for 30 min. Then, the colonies were stained with 0.1% crystal violet for 30 min. After being washed 3 times with PBS, the colonies were counted. The results are presented as the mean values of triplicate samples.

### Scratch wound healing and transwell assays

Scratch wound healing assays were performed on 12-well plates. The cells were plated at 2 × 10^5^ cells/well, and 3 horizontal lines were drawn vertically with a 10 µL pipette tip to ensure that the vertical lines were equal in width. The sloughed cells were washed with PBS. Images were taken at 0 h and 48 h, and the degree of wound healing was measured.

After the cells were digested, they were resuspended in serum-free culture medium. For the migration assay, the density of the cells was 1.5 × 10^5^ cells/mL; for the invasion assay, the density of the cells was 2.5 × 10^5^ cells/mL. For the migration assay, 200 µL of cell mixture was added directly above the transwell chamber. For the invasion assay, 10 µL of Matrigel and 40 µL of prechilled serum-free medium was added to the transwell chamber on ice and incubated for 1 h in a 37 °C incubator. After the Matrigel solidifies, the cell suspension was added. The bottom of the chamber was immersed in medium containing 20% FBS. After 48 h of culture, the chamber was carefully removed with tweezers and washed once in PBS. The cells were fixed in 4% paraformaldehyde for 30 min, washed once in PBS, and then stained with 0.1% crystal violet for 30 min. The remaining cells in the upper chamber were removed. The cells at the bottom of the chamber were photographed with a microscope, and the average number of cells was determined.

### Hoechst/PI assay

The cells were plated on a 96-well plate, and 5 µL of Hoechst 33342 staining solution and 5 µL of PI staining solution (Beyotime Institute of Biotechnology) were added. The cells were then incubated in an ice bath for 30 min and washed once with PBS. Images were taken via a fluorescence microscope, and quantitative analysis was conducted via ImageJ software.

### Cell cycle analysis

The cells were collected and washed with precooled PBS. Then, the cells were precipitated by centrifugation at 1,000 × g for 3–5 min and fixed with cold 70% ethanol overnight at 4 °C. After being washed with precooled PBS, the cells were incubated with 50 µg/mL RNase A solution and 50 µg/mL PI solution for 30 min at 37 °C, and detected by flow cytometry (BD Biosciences, Franklin Lakes, USA). The experimental data were analysed using ModFit software.

### In vivo tumour xenograft model

Four- to six-week-old male BALB/c nude mice were purchased from Vital River Laboratory Animal Technology Co., Ltd. A549 cells stably transfected with shNC (A549-shNC) or shEPN3#2 (A549-shEPN3#2) were injected subcutaneously into the ipsilateral axilla of the mice at a density of 4 × 10^6^ cells per mouse. The length and width of the tumours were measured with a Vernier calliper every three days. In accordance with ethical requirements, all the mice were sacrificed when the largest tumour reached 1,000 mm^3^. The tumours were resected, weighed, and photographed. Some of the tumours were fixed with 4% paraformaldehyde for immunohistochemistry (IHC) and others were used for Western blotting and qPCR.

### In vivo metastatic model

Four- to Six-week-old male BALB/c nude mice were purchased from Vital River Laboratory Animal Technology Co., Ltd. A549 cells stably transfected with shNC (A549-shNC) or shEPN3#2 (A549-shEPN3#2) were injected into the tail veins of the mice. After the injection, the mental state, coat colour, limbs, and behaviour of the nude mice were observed thoroughly. Six weeks later, all the mice were sacrificed. Part of the lung was fixed with 4% paraformaldehyde for IHC and H&E staining, and the other parts were used for Western blotting and qPCR.

### Coimmunoprecipitation (Co-IP)

The plasmid was transfected into the cells. After 48 h, 300 µL of precooled NP-40 buffer (Beyotime Institute of Biotechnology) (containing 1% PMSF) was added to the cells, which were subsequently lysed for 30 min on ice. After centrifugation at 12,000 rpm for 10 min at 4 °C, the cell lysates were incubated with antibodies at 4 °C overnight, followed by incubation with 30 µL of protein A/G magnetic beads (MedChemExpress) for 3 h at 4 °C. After incubation, the beads were washed 6 times with NP-40 buffer and incubated in boiling water for 10 min. The interaction of proteins was confirmed by Western blotting.

### Immunohistochemical analysis

Tumour and lung samples acquired from the mice in the in vivo experiment were fixed with 4% paraformaldehyde for 24 h. Then, the samples were embedded in paraffin and cut into 4-µm sections. After dewaxing, hydration, and antigen retrieval, the sections were incubated in 3% hydrogen peroxide solution at room temperature for 25 min, treated with goat serum at room temperature for 30 min, and then incubated with the primary antibody at 4 °C overnight. The sections were washed 3 times with PBS and incubated with the secondary antibody (Cell Signaling Technology). After the immunostaining was visualized with a diaminobenzidine chromogenic reagent for the histochemical kit (Servicebio Technology Co., Ltd., Wuhan, China), the nuclei were stained with haematoxylin for 3 min. The sections were dehydrated and sealed with glue. The sections were digitally scanned using Nanozoomer Digital Pathology (Hamamatsu Photonics Co., Ltd., Hamamatsu, Japan).

### H&E staining

Lung tissue samples were fixed in 4% paraformaldehyde for 24 h. Then, the samples were embedded in paraffin and cut into sections. After dewaxing and hydration, the sections were stained with haematoxylin and eosin for 5 min each. The sections were dehydrated through ethanol and xylene before being digitally scanned using Nanozoomer Digital Pathology.

### RNA-Seq library preparation and sequencing

TRIzol reagent was used for total RNA extraction from A549-shNC or A549-shEPN3 cells. The RNA concentration and integrity were evaluated by a NanoDrop 2000 (Thermo Fisher Scientific) and an Agilent Bioanalyzer 2100 (DNA Technologies Core, Davis, USA). A VAHTS Universal V6 RNA-seq Library Prep Kit for Illumina (Vazyme Biotech Co., Ltd.) was used for transcriptome library preparation according to the manufacturer’s instructions. The constructed libraries were then subjected to the GenoLab M sequencing platform (GeneMind Biosciences Co., Ltd., Shenzhen, China) in 150-cycle paired-end high-output sequencing mode. The data were analysed by GeneMind Biosciences Co., Ltd.

### Statistical analysis

All of the experimental data are presented as the mean ± standard deviation (SD). Statistical analysis was performed via GraphPad Prism 9.0. Comparisons between groups were performed using unpaired two-tailed Student’s t tests and one-way or two-way analysis of variance (ANOVA) followed by Tukey’s multiple comparisons test, and a *P* value < 0.05 was considered statistically significant.

## Results

### EPN3 is associated with poor prognosis in NSCLC patients

**Fig. 1:**
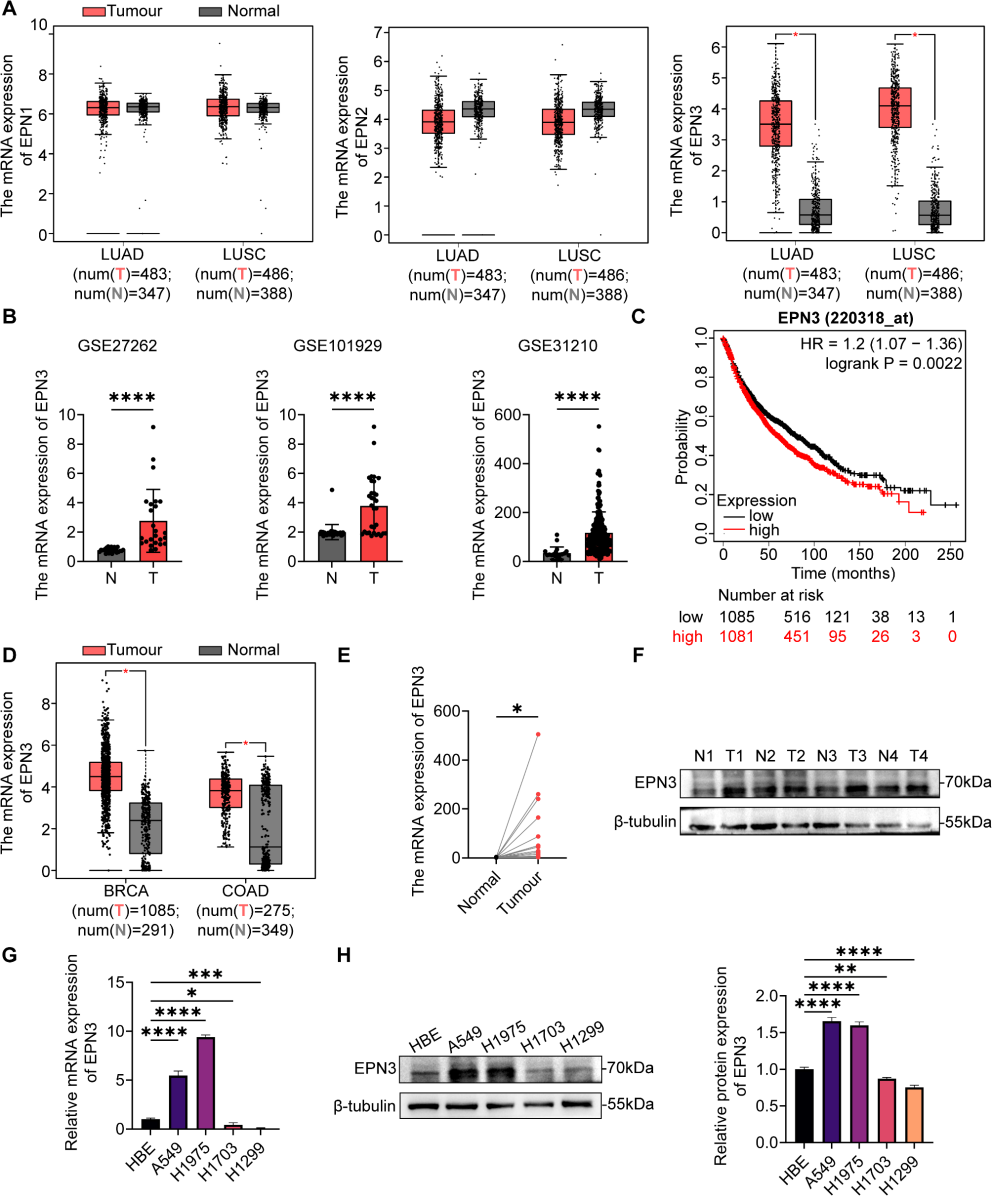
The expression of EPN3 is significantly increased and associated with poor prognosis in patients with NSCLC. **A** TCGA database analysis of EPN1, EPN2 and EPN3 expression levels in LUAD, LUSC and normal tissues. **B** GEO database analysis of EPN3 expression levels in tumour and paracancerous tissue. **C** Kaplan–Meier survival analysis of NSCLC patients stratified according to the EPN3 expression level. **D** TCGA database analysis of EPN3 expression levels in BRCA, COAD and normal tissues. **E** mRNA expression level of EPN3 in 31 paired NSCLC tissues and paracancerous tissues. **F** Protein expression level of EPN3 in paracancerous tissue (N = normal) and lung cancer tissue (T = tumour). **G** mRNA expression level of EPN3 in 16HBE, A549, H1975, H1703 and H1299 cells (*n* = 4). **H** Protein expression level of EPN3 in 16HBE, A549, H1975, H1703 and H1299 cells (*n* = 3). The protein expression levels were normalized to those of β-tubulin in each sample. Data are presented as the mean ± SD. *P < 0.05, **P < 0.01, ***P < 0.001, ****P < 0.0001 vs. control group

To investigate the role of Epsin family proteins in NSCLC, we analysed TCGA genomic data. Compared with that in adjacent tissues, the expression of EPN1 and EPN2 was not significantly different in LUAD and LUSC, whereas EPN3 was significantly highly expressed in LUAD and LUSC (Fig. 1A). EPN3 was also revealed to be highly expressed in NSCLC tissues compared with adjacent normal tissues by analysing datasets from the GEO databases (GSE27262, GSE101929, and GSE31210) (Fig. 1B). Predictive analysis via a website (kmplot.com/analysis/index) indicated that patients with high EPN3 expression had a poor prognosis (Fig. 1C). Additionally, we observed elevated expression of EPN3 in various epithelial malignancies including breast cancer (BRCA) and colon adenocarcinoma (COAD) (Fig. 1D).

Consistently, obviously greater level of EPN3 mRNA expression was detected in 31 pairs of clinical tissue samples by qPCR (Fig. 1E). Moreover, the protein level of EPN3 in NSCLC patient tissues was also markedly increased compared with that in adjacent tissues (Fig. 1F). The mRNA and protein expression levels of EPN3 were subsequently detected in 16HBE, human LUAD A549, H1975, H1299, and human LUSC H1703 cells. Both the mRNA and protein levels of EPN3 were significantly greater in A549 and H1975 cells than in normal lung epithelial cells, whereas H1703 and H1299 cells presented low expression of EPN3 (Fig. 1G, H). Therefore, for subsequent experiments, EPN3 was knocked down in A549 and H1975 cells but overexpressed in H1299 cells.

### Silencing EPN3 inhibits the proliferation of NSCLC cells in vitro

**Fig. 2:**
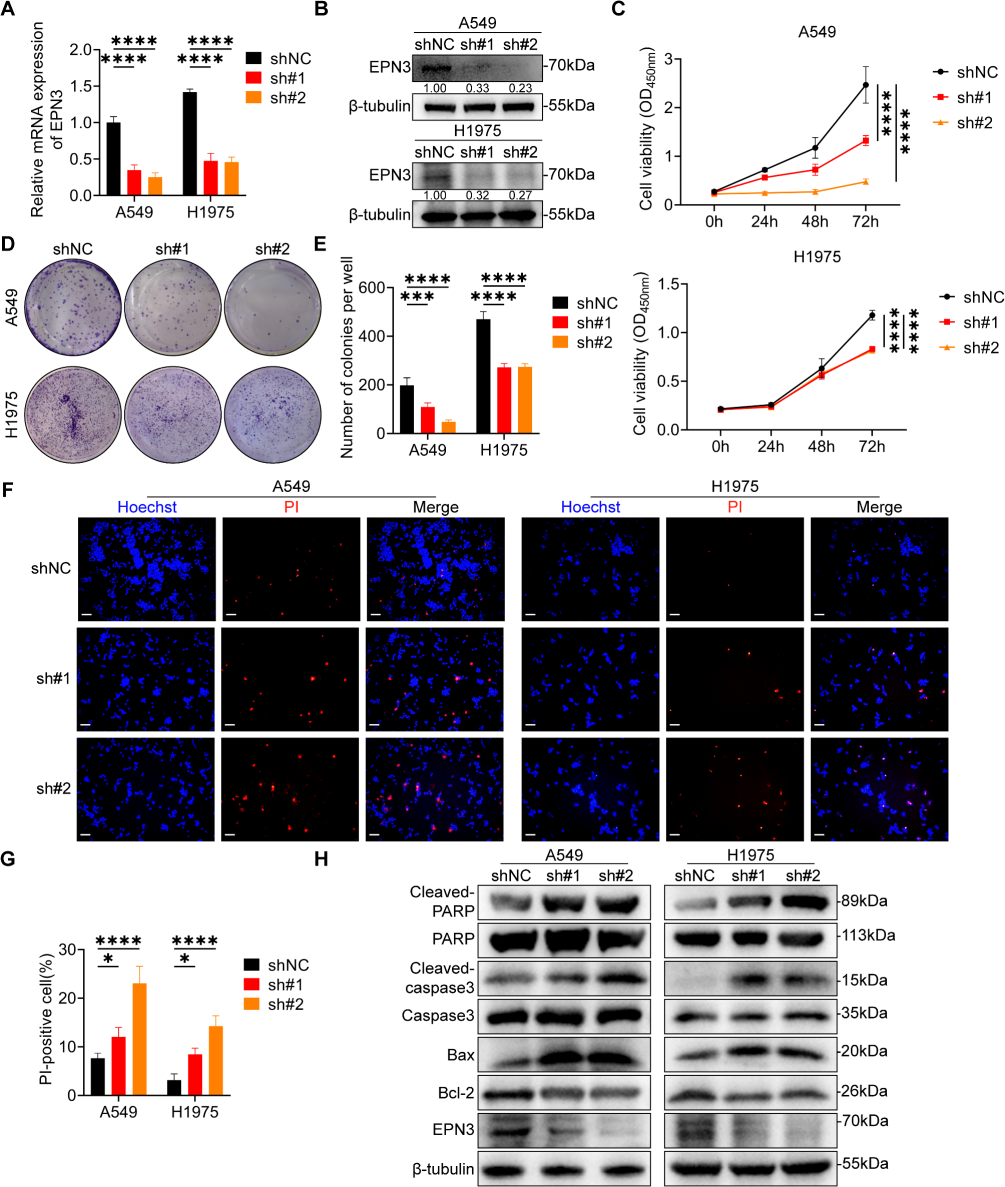
Silencing EPN3 suppresses NSCLC cell proliferation in *vitro*. **A** mRNA expression level of EPN3 in A549 and H1975 cells after EPN3 silencing (*n* = 3). **B** Protein expression level of EPN3 in A549 and H1975 cells after EPN3 silencing (*n* = 3). **C** Quantification of cell viability after EPN3 silencing by a CCK-8 assay (*n* = 5). **D** Colony formation assay to detect colony formation ability after EPN3 silencing. **E** Quantification of colony number after EPN3 silencing (*n* = 3). **F** Representative images of Hoechst/PI staining of A549 and H1975 cells after EPN3 silencing. Scale bar, 100 μm. **G** Quantification of the number of apoptotic cells after EPN3 silencing (*n* = 3). **H** Protein expression levels of cleaved-PARP, PARP, cleaved-caspase 3, caspase 3, Bax, Bcl-2 and EPN3 (*n* = 3). The protein expression levels were normalized to those of β-tubulin in each sample. Data are presented as the mean ± SD. *P < 0.05, ***P < 0.001, ****P < 0.0001 vs. control group

To further investigate the impact of EPN3 on lung cancer cells in vitro. First, A549 and H1975 cell lines in which EPN3 was stably knocked down were constructed by lentivirus (Fig. 2A, B). The results of the CCK-8 assay revealed that EPN3 silencing significantly inhibited the proliferation of A549 and H1975 cells (Fig. 2C). The clonogenic ability of A549 and H1975 cells was markedly reduced by shEPN3 (Fig. 2D, E). These results reveal that EPN3 silencing inhibits the proliferative activity of NSCLC cells in vitro.

Uncontrolled cell proliferation is a characteristic of tumour cells, and is often attributed to a persistent proliferative effect or a reduction in apoptosis [37]. After EPN3 was silenced in A549 and H1975 cells, the distribution of the cell cycle remained unchanged, as detected by flow cytometry (Figure S1A, B). These results suggest that EPN3 is not involved in the cell cycle distribution of NSCLC cells.

We also explored whether EPN3 is associated with the apoptosis of NSCLC cells. Hoechst/PI staining was used to observe cell apoptosis by fluorescence microscopy. The results revealed a significant increase in the number of PI-positive A549 and H1975 cells upon EPN3 knockdown (Fig. 2F, G), suggesting that shEPN3 increased the proportion of apoptotic NSCLC cells. Furthermore, the levels of proteins associated with apoptosis were increased after EPN3 was silenced (Fig. 2H). These results suggest that EPN3 may increase the proliferative capacity of human NSCLC cells by activating the apoptotic pathway.

### Silencing EPN3 inhibits the migration and invasion of NSCLC cells in vitro

**Fig. 3:**
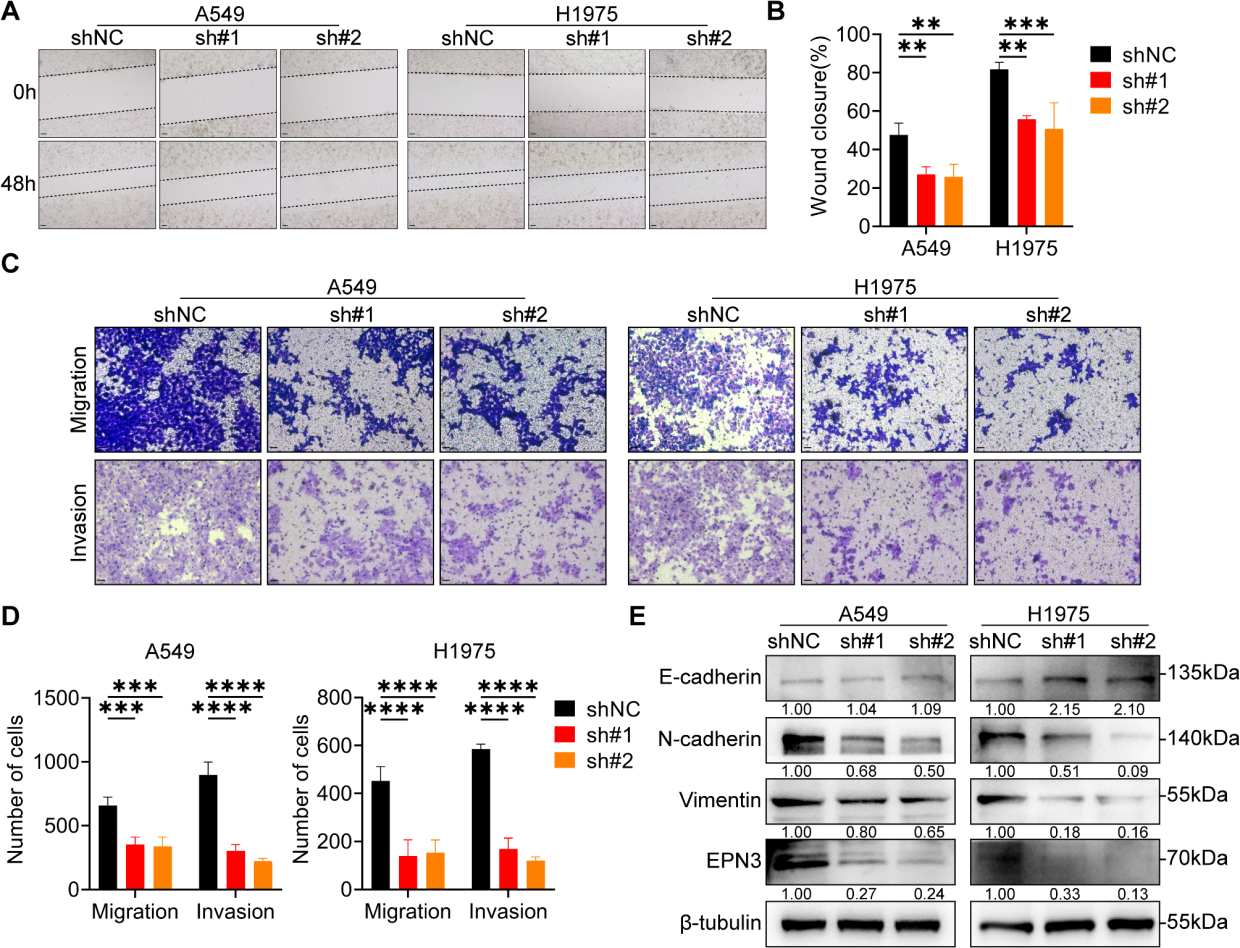
Silencing EPN3 inhibits the migration and invasion of NSCLC cells *in vitro.***A** Scratch wound healing assay investigating the migration ability of A549 and H1975 cells after EPN3 silencing. Scale bar, 100 μm. **B** Quantification of the wound closure area (*n* = 3). **C** Transwell assay evaluating the migration and invasion of A549 and H1975 cells after EPN3 silencing. Scale bar, 100 μm. **D** Quantification of the number of migrated and invaded cells (*n* = 3). **E** Protein expression levels of E-cadherin, N-cadherin, vimentin and EPN3 (*n* = 3). The protein expression levels were normalized to those of β-tubulin in each sample. Data are presented as the mean ± SD. **P < 0.01, ***P < 0.001, ****P < 0.0001 vs. control group

Migration and invasion are key characteristics associated with the incidence of cancer. Compared with the control, the knockdown of EPN3 in both A549 and H1975 cells impaired their migratory and invasive abilities, as demonstrated by the results of the scratch wound healing assay (Fig. 3A, B) and transwell assay (Fig. 3C, D). Epithelial-mesenchymal transition (EMT)-related protein levels were assessed in A549 cells, revealing no significant change in E-cadherin levels with shEPN3, whereas the protein levels of N-cadherin and vimentin were significantly decreased. However, in H1975 cells, the protein level of E-cadherin was significantly increased, and the protein levels of N-cadherin and vimentin were decreased by shEPN3 (Fig. 3E). These results suggest that EPN3 may inhibit the migration and invasion abilities of NSCLC cells by modulating the EMT process.

### Silencing EPN3 inhibits NSCLC proliferation and metastasis in vivo

**Fig. 4:**
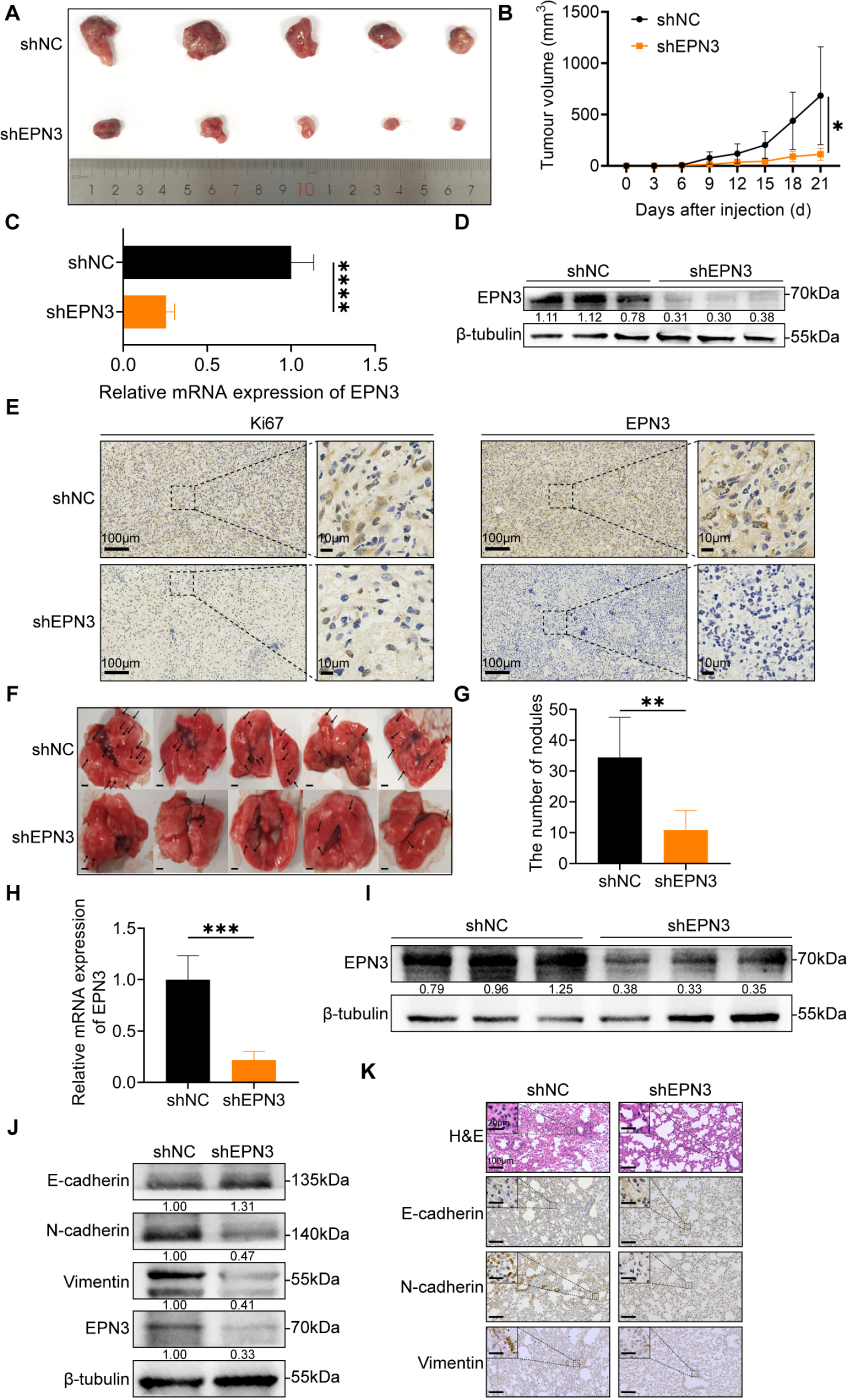
Silencing EPN3 inhibits the proliferation and metastasis of NSCLC cells in *vivo*. **A** A549-shNC and A549-shEPN3 cells were injected into nude mice, the tumours were resected 21 days later, and the tumour volume was visualized in the images. **B** The longest and widest diameters of the tumours were measured every 3 days. The tumour volume (mm^3^) was calculated using the following formula: V (mm^3^) = 1/2×a×b^2^, where a and b represent the longest and widest diameters of the tumour, respectively (*n* = 5). **C** The mRNA expression level of EPN3 in tumours was detected by qPCR (*n* = 4). **D** The protein expression level of EPN3 in tumours was detected by Western blotting (*n* = 3). **E** IHC staining of Ki-67 and EPN3 in serial tumour sections formed from A549-shNC and A549-shEPN3 cells. **F** Representative lung images of nude mice injected with A549-shNC or A549-shEPN3 cells via the tail vein. The black arrows indicate representative lung nodules. Scale bar, 2 mm. **G** Quantification of the lung nodules (*n* = 5). **H** The mRNA expression level of EPN3 in lung tissue was detected by qPCR (*n* = 4). **I** The protein expression level of EPN3 in lung tissue was detected by Western blotting (*n* = 3). **J** The expression levels of EMT-related proteins in lung tissue were detected by Western blotting (*n* = 3). **K** Representative images of lung tissue sections and H&E staining after mouse tail vein injection of A549 cells after EPN3 silencing. The protein expression levels were normalized to those of β-tubulin in each sample. Data are presented as the mean ± SD. *P < 0.05, **P < 0.01, ***P < 0.001, ****P < 0.0001 vs. control group

To evaluate the impact of EPN3 on the proliferative ability of NSCLC tumours in *vivo*, A549-shNC and A549-shEPN3 cells were injected into the ipsilateral armpit of male BALB/c nude mice. After 21 days, the growth rate of tumours was significantly lower in the mice injected with EPN3-knockdown cells than in those injected with A549-shNC cells (Fig. 4A, B). The efficiency of EPN3 knockdown was verified by qPCR and Western blotting (Fig. 4C, D). As shown in Fig. 4E, EPN3 silencing resulted in a decreased number of Ki67-positive cells in the xenograft mouse tumours. Collectively, these findings indicate that EPN3 is necessary for the proliferation of lung cancer cells.

To further investigate whether EPN3 also affects the metastasis of NSCLC cells in *vivo*, A549-shNC and A549-shEPN3 cells were injected into the tail veins of male BALB/c nude mice. Six weeks later, the number of metastatic nodules on the lung surface was significantly lower in the mice injected with A549-shEPN3 cells than in those injected with A549-shNC cells (Fig. 4F, G). Additionally, compared with mice injected with A549-shEPN3 cells, those injected with A549-shNC cells presented more severe liver injury (Figure S2A). EPN3 expression was verified by qPCR and Western blotting (Fig. 4H, I). Furthermore, the protein levels of E-cadherin were significantly increased, whereas those of N-cadherin and vimentin were significantly decreased in the lungs of nude mice injected with A549-shEPN3 cells, which was consistent with the immunohistochemistry results (Fig. 4J, K). H&E staining revealed a relative reduction in lung metastatic nodules on the lung surface of nude mice injected with A549-shEPN3 cells, with a relatively normal pulmonary vacuolar structure (Fig. 4K). These results indicate that silencing EPN3 significantly inhibits the metastasis of NSCLC cells *in vivo.*

### EPN3 enhances EGFR stability and signalling activity by inhibiting the EGFR lysosomal degradation pathway

**Fig. 5:**
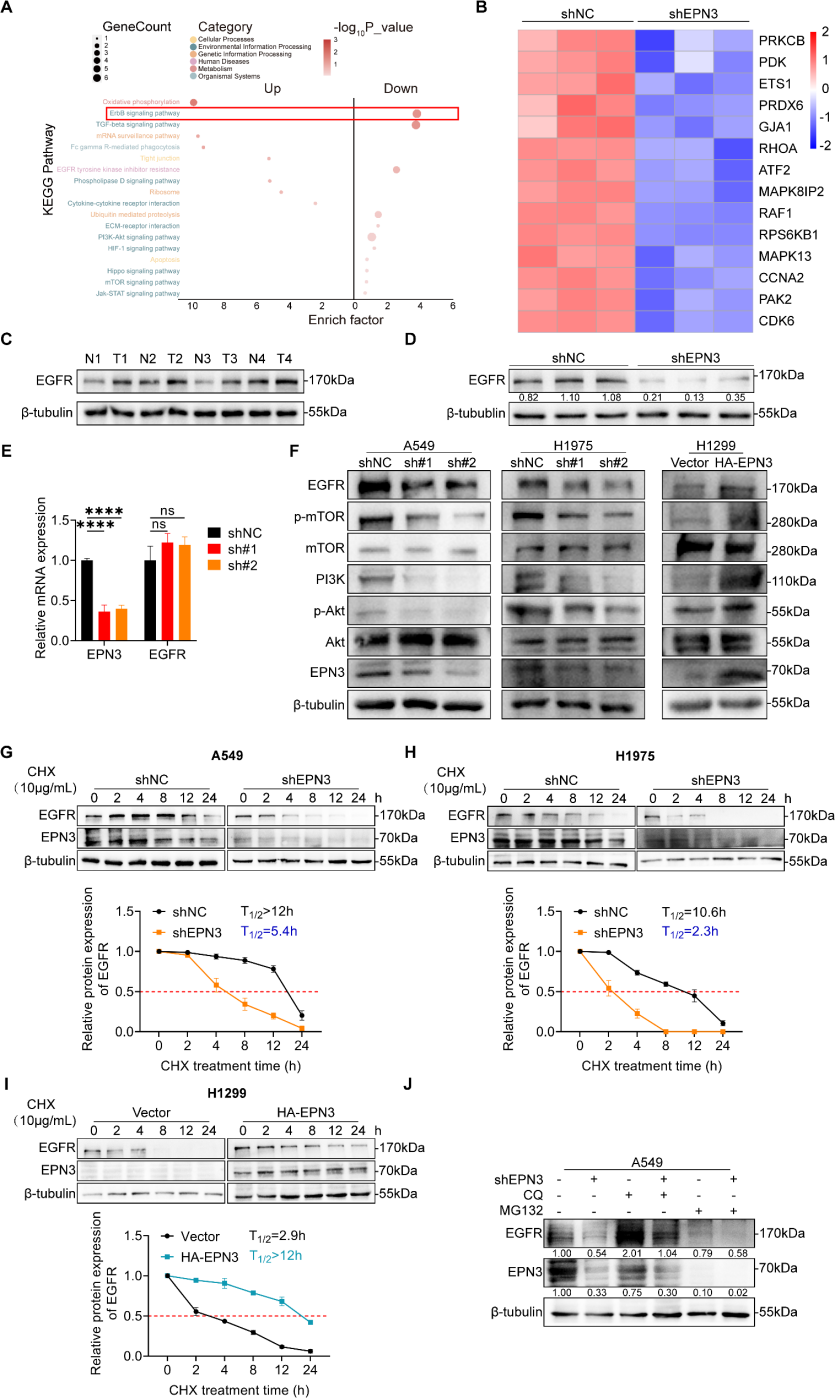
EPN3 enhances EGFR stability and signalling activity by inhibiting the EGFR lysosomal degradation pathway. **A** KEGG enrichment analysis of A549 cells in which EPN3 was silenced compared with the NC group. **B** Heatmap showing different gene expression levels in the ErbB signalling pathway. **C** The protein expression level of EGFR in paracancerous tissue (N = normal) and lung cancer tissue (T = tumour). **D** The protein expression level of EGFR in tumours was detected by Western blotting (*n* = 3). **E** The mRNA expression level of EGFR was detected by qPCR (*n* = 3). **F** Protein expression levels of EGFR and downstream effector proteins in A549 and H1975 cells with silenced EPN3 or in H1299 cells overexpressing EPN3. **G** A549-shNC or A549-shEPN3 cells were treated with CHX (10 µg/ml) at the indicated intervals. The protein stability of EGFR was analysed by Western blotting. Quantitative analysis of the EGFR protein half-life (*n* = 3). **H** H1975-shNC cells or H1975-shEPN3 cells were treated with CHX (10 µg/ml) at the indicated intervals. The protein stability of EGFR was analysed by Western blotting. Quantitative analysis of the EGFR protein half-life (*n* = 3). **I** Control or EPN3-overexpressing H1299 cells were treated with CHX (10 µg/ml) at the indicated intervals. The protein stability of EGFR was analysed by Western blotting. Quantitative analysis of the EGFR protein half-life (*n* = 3). **J** The lysosomal inhibitor CQ (50 µM) and the proteasome inhibitor MG132 (10 µM) were added to A549-shNC or A549-shEPN3 cells. The protein expression levels of EGFR and EPN3 were analysed by Western blotting (*n* = 3). The protein expression levels were normalized to those of β-tubulin in each sample. Data are presented as the mean ± SD. ns indicates not significant, ****P < 0.0001 vs. control group

To elucidate the mechanism by which EPN3 regulates lung cancer growth and metastasis, we performed RNA-seq in A549 cells with stable EPN3 knockdown. Pathway analysis revealed significant enrichment of the ErbB signalling pathway (Fig. 5A), and heatmap analysis demonstrated that EPN3 influenced the transcription of ErbB signalling pathway-related genes (Fig. 5B). Moreover, the increased EGFR protein levels in the tumour tissues of NSCLC patients and the decreased EGFR protein levels in xenograft tumours following EPN3 knockdown suggest that the ErbB signalling pathway may play a role in A549 cell proliferation and differentiation after EPN3 knockdown (Fig. 5C, D). The critical role of EGFR (ErbB1) activation in cell proliferation and differentiation explains its direct relationship with human cancer development. Notably, EPN3 silencing did not impact EGFR transcription in A549 cells (Fig. 5E). The expression levels of EGFR and its downstream effector proteins were subsequently measured after EPN3 was silenced in A549 and H1975 cells or after EPN3 was overexpressed in H1299 cells. A549 and H1299 cells harbour WT-EGFR, whereas H1975 cells are TKI-resistant cells that harbour the L858R/T790M double mutant EGFR [38, 39]. Moreover, knockdown of EPN3 in both cell types was conducted to validate whether EGFR mutations influence the regulatory role of EPN3 in the EGFR downstream pathway. The results demonstrated that EGFR protein expression was reduced by EPN3 knockdown in both A549 and H975 cells, along with the p-mTOR, PI3K and p-AKT protein levels were also decreased. Conversely, the level of EGFR and downstream proteins were increased by EPN3 overexpression (Fig. 5F). These findings indicate that EPN3 modulates the protein level but not the mRNA level of EGFR, suggesting that posttranscriptional regulation influences downstream EGFR signalling, which is unaffected by the EGFR mutation status.

Next, we investigated whether EPN3 regulates EGFR stability. In A549 cells with EPN3 knockdown, the half-life of the endogenous EGFR protein was markedly shorter in the presence of cycloheximide (CHX), a protein synthesis inhibitor (Fig. 5G). A similar reduction in the EGFR half-life was observed in H1975 cells expressing EGFR with L858R/T790M double mutations following EPN3 silencing (Fig. 5H). Concomitantly, a prolonged half-life of EGFR was observed in H1299 cells following EPN3 overexpression (Fig. 5I). Thus, these data suggest that EPN3 positively regulates the stability of EGFR, whether it is WT-EGFR or its mutants.

A previous study highlighted two protein degradation systems targeting lysosomes and proteasomes for EGFR [40], and EGFR stability is closely related to tumour occurrence and development [41]. To confirm the involvement of EPN3 in EGFR degradation, the lysosomal inhibitor CQ and the proteasome inhibitor MG132 were used in A549 cells. The results revealed that the EGFR protein obviously accumulated in cells treated with CQ but not MG132 (Fig. 5J), indicating that EPN3 influences EGFR stability by inhibiting the lysosomal degradation pathway.

### EPN3 interacts with EGFR to promote EGFR membrane recycling

**Fig. 6:**
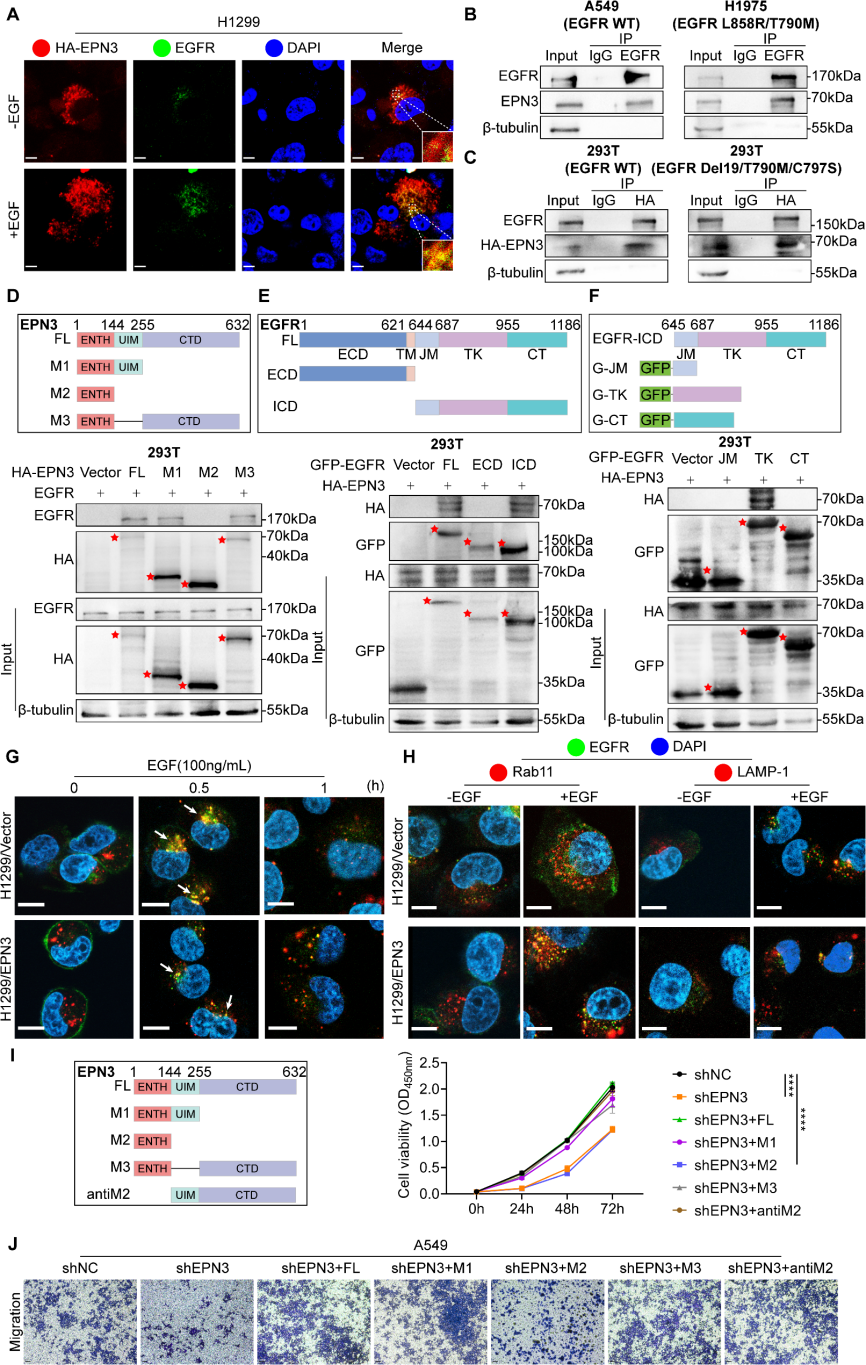
EPN3 interacts with EGFR to promote EGFR membrane recycling. **A** Confocal microscopy images showing the distribution of EPN3 (red) and EGFR (green) in H1299 cells stimulated with or without EGF (100 ng/mL) for 30 min. Scale bar, 5 μm. **B** The interaction between the EGFR and EPN3 proteins was detected by Co-IP in A549 and H1975 cells. **C** WT-EGFR, mutant EGFR (Del19/T790M/C797S) and EPN3 were overexpressed in 293T cells, and the interaction between EPN3 and EGFR was detected via Co-IP. **D** Schematic diagram of the HA-EPN3 deletion mutants (top). 293T cells were co-transfected with WT-EGFR and HA-EPN3 deletion mutant plasmids. The EPN3 region involved in the EGFR interaction was analysed by Co-IP (bottom). **E** Schematic diagram of the GFP-EGFR deletion mutants (top). 293T cells were cotransfected with HA-EPN3 and GFP-EGFR deletion mutant plasmids. The EGFR region involved in the EPN3 interaction was analysed by Co-IP (bottom). **F** Schematic diagram of GFP-EGFR-ICD deletion mutants (top). 293T cells were cotransfected with HA-EPN3 and GFP-ICD deletion mutant plasmids. The EGFR-ICD region involved in the EPN3 interaction was analysed by Co-IP (bottom). **G** Confocal microscopy images showing the distribution of EGFR (green) and early endosome antigen 1 (red) after EGF (100 ng/mL) stimulation for 0.5–1 h in control H1299 cells and H1299 cells overexpressing EPN3. Scale bar, 10 μm. **H** Confocal microscopy images showing the colocalization of EGFR (green) with Rab11 (red) or LAMP-1 (red) after 30 min of EGF treatment in control H1299 cells and H1299 cells overexpressing EPN3. Scale bar, 10 μm. **I** Schematic diagram of the construction of HA-EPN3 deletion mutants (left). CCK-8 assay measuring cell viability after the transfection of HA-EPN3 deletion mutant plasmids in A549-shEPN3 cells (right) (*n* = 5). **J** Transwell assay in A549-shEPN3 cells after transfection with HA-EPN3 deletion mutant plasmids (*n* = 3). Data are presented as the mean ± SD. ****P < 0.0001 vs. control group

To further investigate the posttranslational regulatory effect of EPN3 on EGFR, an immunofluorescence experiment was performed. HA-tagged EPN3 colocalized with both endogenous and exogenous EGFR in the cytoplasm (Fig. 6A and Figure S3A). Co-IP assays further revealed that EPN3 precipitated with EGFR in both A549 and H1975 cells (Fig. 6B). The interaction between EPN3 and EGFE was subsequently validated through Co-IP in HEK-293T cells transfected with HA-EPN3 and WT-EGFR or the EGFR Del19/T790M/C797S triple mutant plasmid, which revealed that exogenous EPN3 could interact with both WT-EGFR and EGFR with a third-generation EGFR-TKI resistant mutant (Del19/T790M/C797S) (Fig. 6C).

To confirm the interaction region of EPN3 with EGFR, deletion mutants of HA-tagged EPN3 plasmids were constructed and subjected to a Co-IP assay (Fig. 6D, top). In 293T cells, the results revealed that the ubiquitin-interacting motif (UIM) region and C-terminal domain (CTD) region of EPN3 interact with EGFR (Fig. 6D, bottom). Additionally, EGFR was divided into two parts on the basis of the ECD and ICD, and the ICD was found to be responsible for the association with EPN3 (Fig. 6E). Furthermore, the intracellular JM region, TK region, and CT region of the plasmids were constructed according to the functional distribution of the EGFR intracellular region (Fig. 6F, top), the results showed that TK region of EGFR interacts with EPN3 (Fig. 6F, bottom). Therefore, these data indicate that the UIM and CTD regions of EPN3 interact with the TK region of EGFR to regulate its stability and signalling activity.

We conducted a more thorough investigation into the influence of the interaction between EPN3 and EGFR on EGFR recycling. Under EGF stimulation, EGFRs are internalized into early endosomes, and these EGFRs may return to the cell surface through circulating bodies, while those not entering circulating bodies undergo degradation [42]. EGF stimulation did not affect the entry of EGFR into early endosomes in normal H1299 cells or H1299-overexpressing EPN3 cells after 30 min of EGF stimulation. However, with prolonged EGF stimulation for 60 min, there was a relative increase in EGFR levels in cells overexpressing EPN3 (indicated by white arrows) (Fig. 6G). These findings suggest that EPN3 can affect EGFR protein levels on the cell membrane. Moreover, a substantial amount of EGFR predominantly colocalized with Rab11-positive recycling vesicles in H1299-overexpressing EPN3 cells after EGF stimulation, whereas the colocalization of EGFR with lysosome-associated membrane protein 1 (LAMP1, a lysosomal marker) was much less frequent (Fig. 6H). These results suggest that high expression of EPN3 facilitates the entry of a significant amount of EGFR into the circulation, reducing its lysosomal degradation. EPN3 promotes EGFR cycling through protein interactions, enhancing EGFR stability.

To verify whether the proliferation and migration ability of tumour cells can be reversed by the upregulation of EPN3, EPN3 was overexpressed in EPN3-silenced A549 cells. Additionally, to determine whether the ability of EPN3 to regulate the proliferation and metastasis of NSCLC cells depends on its structural integrity, EPN3-silenced A549 cells were transfected with full-length HA-tagged EPN3 and HA-tagged EPN3 deletion mutants (Fig. 6I, left). The results revealed that the transfection of full-length EPN3 and the M1, M3, and anti-M2 fragment plasmids into EPN3-silenced A549 cells reversed the increase in the cell proliferation rate, whereas the viability of the cells transfected with the M2 fragment plasmids was still significantly reduced (Fig. 6I, right). The transwell assay results revealed that the number of migrated cells increased significantly when EPN3-silenced A549 cells were transfected with full-length EPN3, M1, M3 or anti-M2 fragment plasmids, whereas the number of migrated cells was significantly inhibited when EPN3-silenced A549 cells were transfected with M2 fragment plasmids (Fig. 6J). Therefore, restoring EPN3 expression in EPN3-silenced cell lines can rescue the ability of EPN3 to regulate the proliferation and metastasis of NSCLC cells, and its regulatory ability depends on the UIM region or CTD region where EPN3 interacts with EGFR.

## Discussion

In this study, consistent with previous reports, we identified EPN3, a member of the adaptor protein family involved in receptor endocytosis, as a cancer marker in NSCLC, and its upregulation was correlated with NSCLC progression. Given the established role of EPN3 in endocytosis-related pathways, we aimed to elucidate its specific mechanisms in NSCLC. Previous studies have indicated that EGF stimulation induces the ubiquitination of EGFR, followed by its interaction with EPN1 [43]. Epsins facilitate EGFR recruitment into clathrin-coated pits for internalization. Within the clathrin-mediated endocytic pathway and during early endosome trafficking, EGFR kinase activity remains intact [40]. The interaction between Epsin and EGFR significantly alters EGF signalling, with evidence also showing that GSDME stabilizes EGFR by shielding the Y1045 site [44]. Knockout of AP1S1 leads to lysosomal degradation of EGFR and increases sensitivity to erlotinib-resistant H1975 cells [39]. Moreover, through RNA-seq and database analysis, we identified a positive correlation between EPN3 and EGFR. Therefore, we focused on EPN3 promoting NSCLC by regulating the turnover of EGFR. Our findings confirmed that EPN3 interacts with EGFR and elevated EPN3 expression significantly stabilizes EGFR by inhibiting its lysosomal degradation. This stabilization prolongs the half-life of EGFR, promotes its recycling to the membrane, and enhances downstream signalling pathways, all of which are critical for NSCLC progression.

Although targeted EGFR therapies such as TKIs have shown substantial efficacy in the treatment of EGFR-mutant NSCLC, the emergence of TKI resistance in patients remains a significant obstacle [45]. Mutations in the TK domain of EGFR play pivotal roles in both EGFR-TKI resistance and the progression of NSCLC [9, 46]. Given the crucial role of EGFR in NSCLC and the clinical efficacy of EGFR-targeted therapies such as TKIs, our findings offer a novel perspective on the mechanisms underlying TKI resistance. Our findings suggest that EPN3 may sustain EGFR signalling even in the presence of such mutations, potentially contributing to TKI resistance. If the interaction between EPN3 and EGFR can be interfered with by designing peptides through the binding site and the degradation of EGFR can be promoted, this may present a promising avenue for clinical treatment and drug development in NSCLC, particularly for TKI-resistant patients.

During the research process of this study, we discovered that silencing EPN3 downregulated the protein levels of EGFR and its downstream signals, with a more pronounced decrease in PI3K protein levels compared to EGFR. In this regard, we reviewed relevant literature and found that EPN3 not only exerts oncogenic effects in NSCLC by regulating the EGFR signalling pathway but also exerts similar effects by activating the JAK1/2-STAT3 pathway [34]. The JAK pathway is closely associated with the PI3K/AKT/mTOR pathway, and its inhibition can significantly reduce PI3K/AKT/mTOR pathway activity [47, 48]. Furthermore, the TGF-β signalling pathway is also capable of activating the PI3K/AKT/mTOR pathway [49, 50]. RNA-seq data provided additional support for this hypothesis, demonstrating that the TGF-β signalling pathway was inhibited in A549 cells following EPN3 silencing (Fig. 5A). These findings suggest that EPN3 silencing may modulate the PI3K/AKT/mTOR pathway not only via the EGFR signalling pathway but also through the JAK or TGF-β signalling pathways, leading to a more substantial impact on PI3K protein levels. However, this hypothesis requires detailed validation in future studies. It also remains unclear whether EPN3 silencing influences other oncogenic pathways, such as c-MET, HER2, IGFR or AXL, underscoring the need for further investigation [51, 52]. Moreover, as a key regulatory pathway in lung cancer cell growth, the EGFR signalling pathway can amplify signals through downstream cascade reactions, potentially resulting in more pronounced effects on downstream proteins compared to EGFR itself. In addition, although the EPN3 protein was silenced and overexpressed in this study and the specific binding relationship between EPN3 and the EGFR protein was studied through the construction of a deletion mutant body, the exact amino acids responsible for these interactions remain unclear. Research can also further explore whether EPN3 can affect the regulation of EGFR through key amino acid point mutations.

This study revealed that EPN3 affects the stability of the EGFR protein by inhibiting the EGFR lysosomal degradation pathway. Ubiquitination has long been recognized for its role in protein degradation, but emerging evidence suggests that it also plays a role in signal transduction, transcriptional regulation, and protein recycling, particularly through K63-linked polyubiquitination [53]. Additionally, the involvement of EPN3 in EGFR endocytosis appears to depend on the integrity of its UIM [54]. Although we determined that EPN3 is a key factor in regulating EGFR stability, its specific molecular mechanism still requires in-depth study. The specific ubiquitinated proteins involved in the EPN3-EGFR interaction remain to be elucidated. In future studies, the ubiquitinated proteins affecting the ubiquitin‒lysosomal degradation pathway of EGFR could serve as crucial research targets to further understand the endocytosis and transport process of EGFR. Furthermore, the specific ubiquitination sites of EGFR related to EPN3-EGFR binding also require further exploration.

## Conclusion

**Fig. 7:**
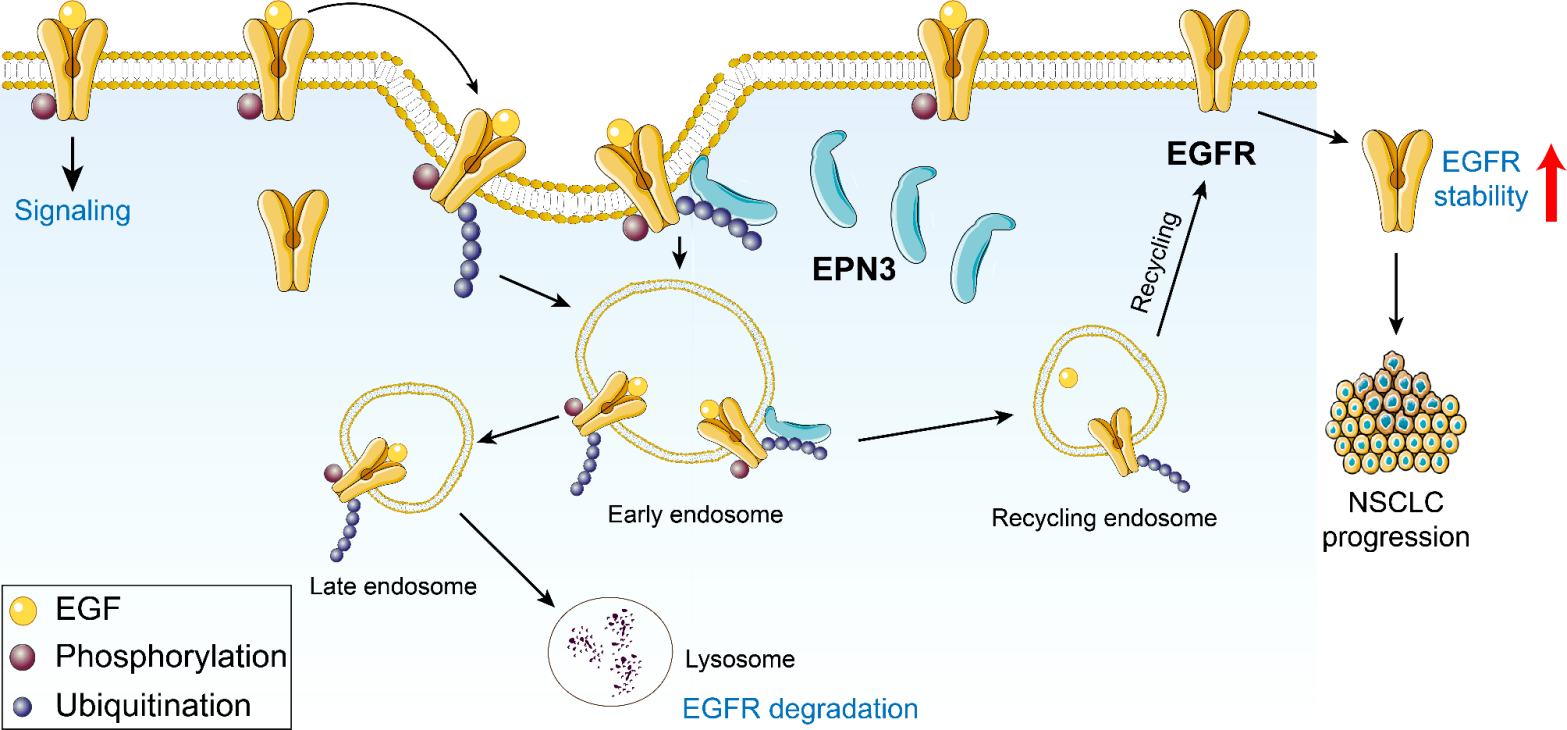
Graphical abstract image illustrating the mechanism of EPN3-mediated EGFR stability and its role in NSCLC development. EPN3 interacts with EGFR, inhibiting the lysosomal degradation pathway of EGFR, enhancing the membrane recycling of EGFR and increasing EGFR stability and downstream signalling activity, thereby promoting the progression of NSCLC

In summary, we concluded that the interaction of EPN3 with EGFR inhibits the EGFR-targeted lysosomal degradation pathway, promoting EGFR entry into the endocytic recycling system, increasing membrane recycling, increasing EGFR stability, and increasing downstream signalling, all of which contribute to NSCLC progression (Fig. 7). Our study suggests that EPN3 is a promising therapeutic target in NSCLC, especially in patients with TKI-resistant EGFR mutations. By inhibiting EPN3, it promotes EGFR degradation, reduces signalling activity, and overcomes resistance to EGFR-targeted therapies. This study provides a solid theoretical basis for the development of new drugs targeting EPN3 and provides a new approach for the treatment of NSCLC.

## Electronic supplementary material

Below is the link to the electronic supplementary material.

Supplementary Material 1: Supplementary Figure 1. Silencing EPN3 does not affect the cell cycle distribution of NSCLC cells.

Supplementary Material 2: Supplementary Figure 2. Silencing EPN3 inhibits the liver metastasis of NSCLC cells.

Supplementary Material 3: Supplementary Figure 3 Exogenous EPN3 and EGFR were colocalized in NSCLC cells.

## Data Availability

All microarray data generated in this study have been deposited at the NCBI Gene Expression Omnibus with the accession codes GSE27262, GSE31210 and GSE101929. Correlations between EGFR and EPN3 mRNA expression across TCGA lung cancer datasets were analysed via the following website: http://gepia.cancer-pku.cn. The KM plotter lung cancer dataset was obtained from http://kmplot.com/analysis. The RNA-seq data that support the findings of this study have been deposited into the CNGB Sequence Archive (CNSA) of the China National GeneBank DataBase (CNGBdb) with accession number CNP0004492. All other data supporting the findings of this study are available from the corresponding authors upon reasonable request. Source data are provided with this paper.

## Abbreviations

BRCA: Breast cancer
CHX: Cycloheximide
COAD: Colon adenocarcinoma
Co-IP: Coimmunoprecipitation
CQ: Chloroquine
CT: C-terminal
CTD: C-terminal domain
DMEM: Dulbecco’s modified Eagle’s medium
ECD: Extracellular domain
EGF: Epidermal growth factor
EGFR: Epidermal growth factor receptor
EMT: Epithelial–mesenchymal transition
EPN1: Epsin1
EPN2: Epsin2
EPN3: Epsin3
FBS: Foetal bovine serum
GFP: Green fluorescent protein
ICD: Intracellular domain
IHC: Immunohistochemical
JM: Juxtamembrane
LAMP1: Lysosome-associated membrane protein 1
LUAD: Lung adenocarcinoma
LUSA: Lung squamous cell carcinoma
NC: Negative control
NSCLC: Non-small cell lung cancer
PI: Propidium iodide
qPCR: Quantitative real-time polymerase chain reaction
TK: Tyrosine kinase
TKI: Tyrosine kinase inhibitor
UIM: Ubiquitin-interacting motif
WT: Wild type
