# Supplementary material for: Epsin3 promotes non-small cell lung cancer progression via modulating EGFR stability
Source: Cell Biosci. 2025 Feb 5;15:14. doi: 10.1186/s13578-025-01358-1 (PMC11800460; doi:10.1186/s13578-025-01358-1)
Supplement: Supplementary file 1 — Supplementary Material 1: Supplementary Figure 1. Silencing EPN3 does not affect the cell cycle distribution of NSCLC cells. [file 13578_2025_1358_MOESM1_ESM.doc]

**Supplementary figures**

**
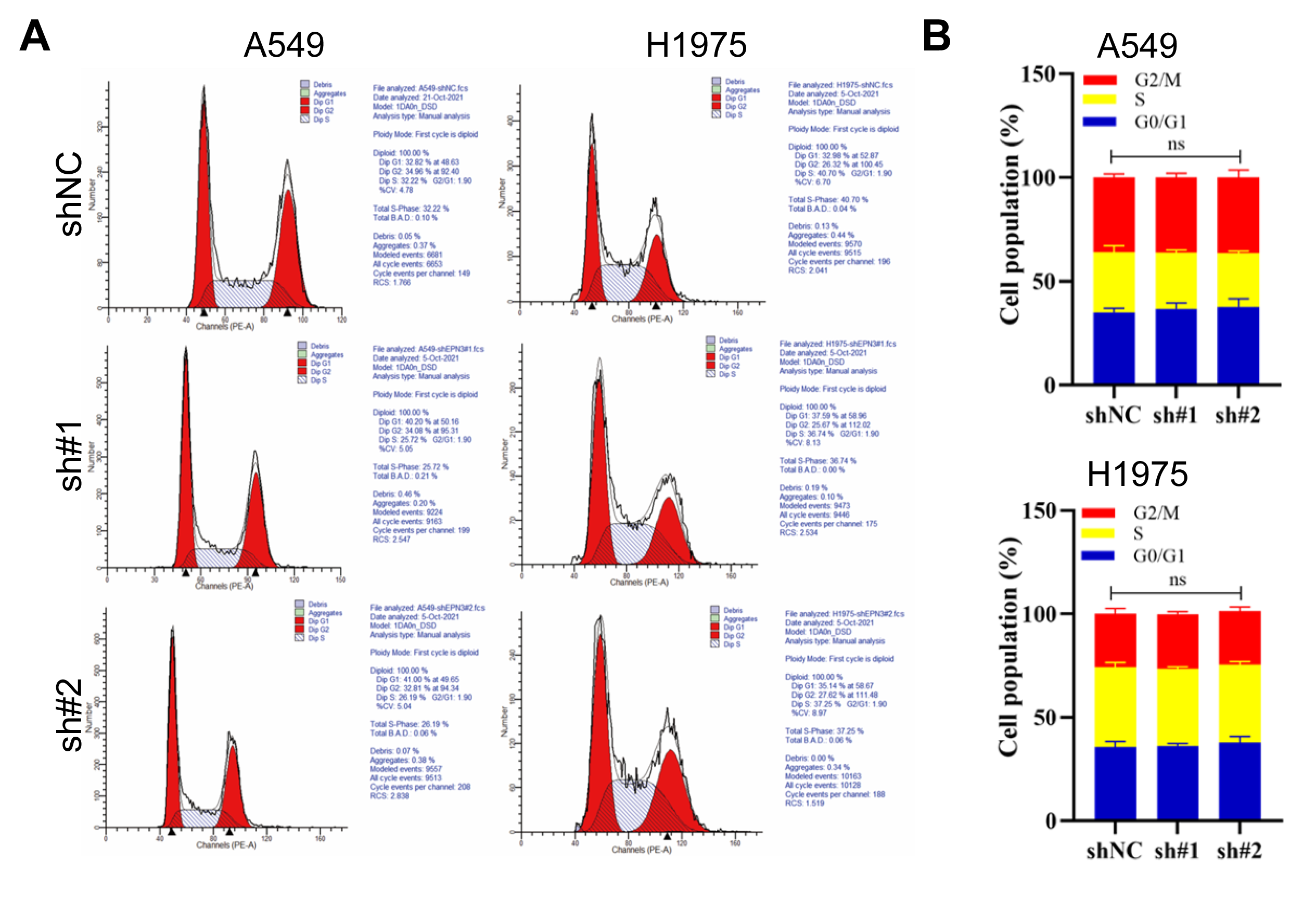
**

**Supplementary Figure 1** Silencing EPN3 does not affect the cell cycle distribution in NSCLC cells. **A** The cell cycle distribution in A549 and H1975 cells after EPN3 silencing detected by flow cytometry. **B** Quantification of A549 and H1975 cells at each stage (n=3). ns indicates not significant.


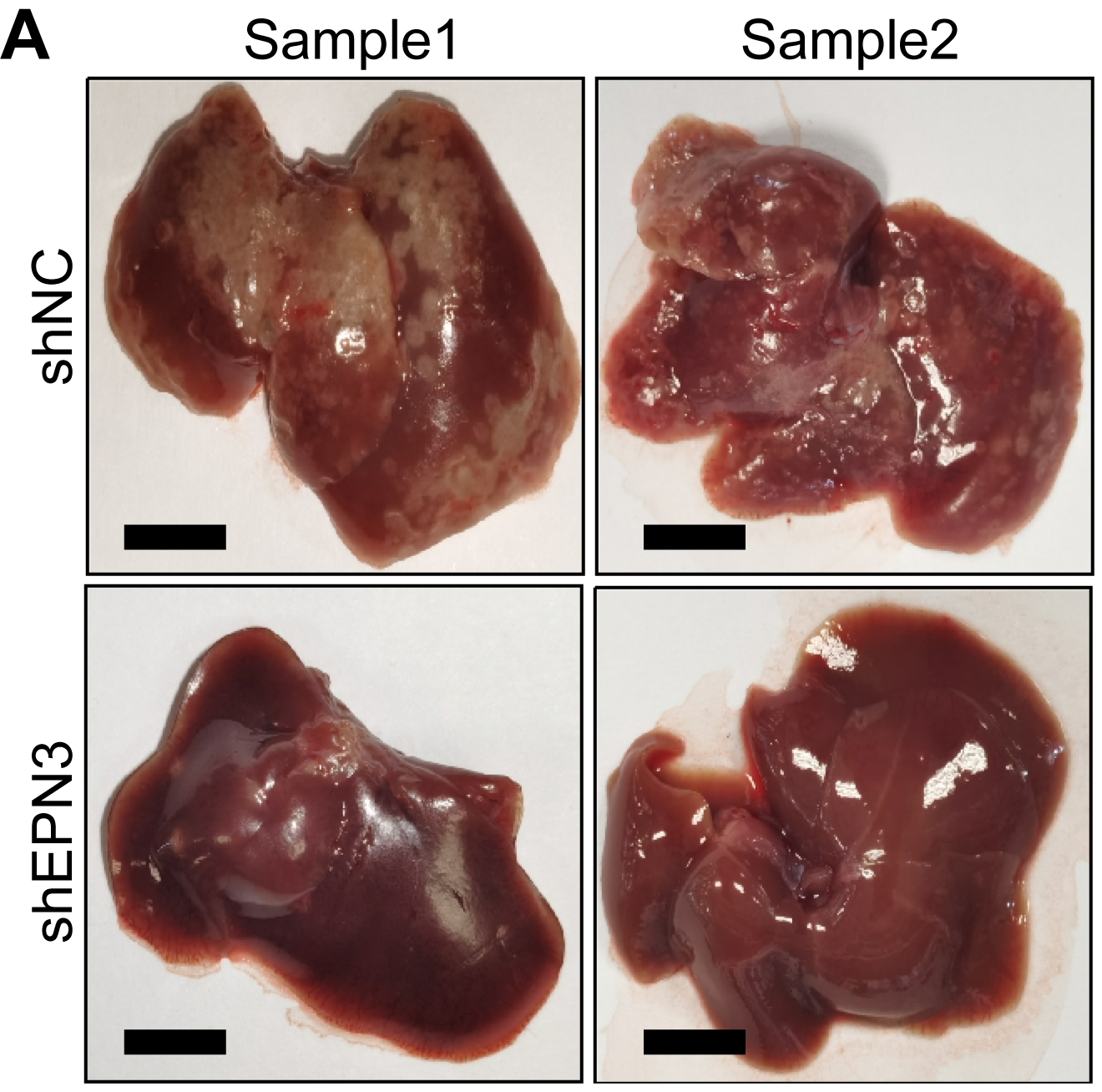


**Supplementary Figure 2** Silencing EPN3 inhibits the liver metastasis of NSCLC cells. **A** Representative image of the liver tissues after mouse tail vein injection of A549-shNC or A549-shEPN3 cells. Scale bar, 5 mm.


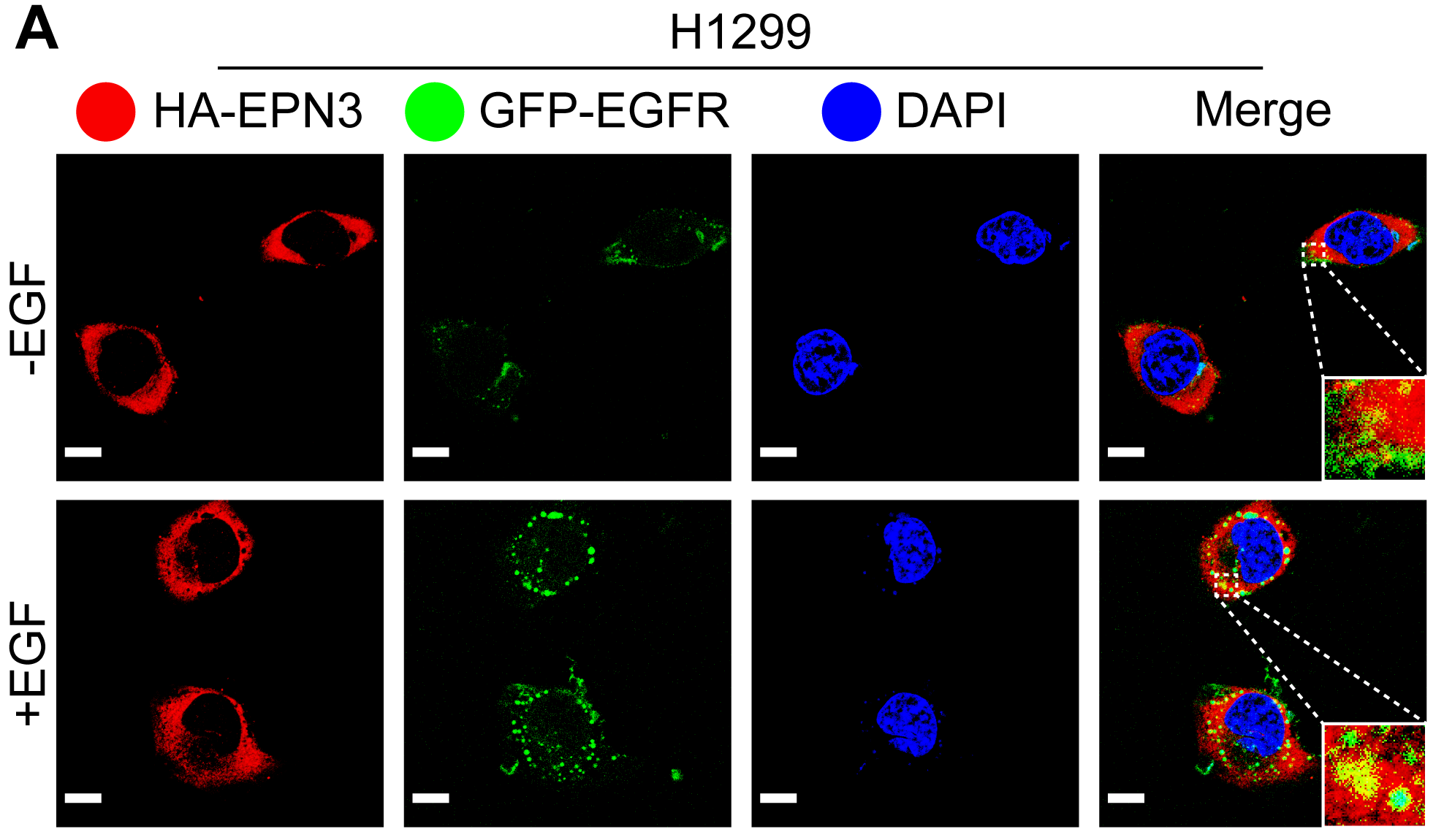


**Supplementary Figure 3** Exogenous EPN3 and EGFR were colocalized in NSCLC cells. **A** Confocal microscopy images showing the distribution of HA-EPN3 (red) and GFP-EGFR (green) in H1299 cells stimulated with or without EGF (100 ng/mL) for 30 min. Scale bar, 5 μm.
