# Supplementary material for: Epsin3 promotes non-small cell lung cancer progression via modulating EGFR stability
Source: Cell Biosci. 2025 Feb 5;15:14. doi: 10.1186/s13578-025-01358-1 (PMC11800460; doi:10.1186/s13578-025-01358-1)
Supplement: Supplementary file 2 — Supplementary Material 2: Supplementary Figure 2. Silencing EPN3 inhibits the liver metastasis of NSCLC cells. [file 13578_2025_1358_MOESM2_ESM.pdf]

Editing Certificate

This document certifies that the manuscript

Epsin3 promotes non-small cell lung cancer progression via modulating EGFR stability

prepared by the authors

Huiling Su, Jie Shen, Chenzi Gao, Yue Zhao, Wanyu Deng, Bo Qin, Xin Zhang, Juan Lai, Qian Wang, Jie Dou, Min Guo

was edited for proper English language, grammar, punctuation, spelling, and overall style by one or more of the highly qualified English speaking editors at AJE.

This certificate was issued on **December 16, 2024** and may be verified on the AJE website using the verification code **02B1-FB29-0EFB-F612-533B** .

Neither the research content nor the authors' intentions were altered in any way during the editing process. Documents receiving this certification should be English-ready for publication; however, the author has the ability to accept or reject our suggestions and changes. To verify the final AJE edited version, please visit our verification page at aje.com/certificate. If you have any questions or concerns about this edited document, please contact AJE at support@aje.com.

AJE provides a range of editing, translation, and manuscript services for researchers and publishers around the world. For more information about our company, services, and partner discounts, please visit aje.com.
