# Supplementary material for: Epsin3 promotes non-small cell lung cancer progression via modulating EGFR stability
Source: Cell Biosci. 2025 Feb 5;15:14. doi: 10.1186/s13578-025-01358-1 (PMC11800460; doi:10.1186/s13578-025-01358-1)

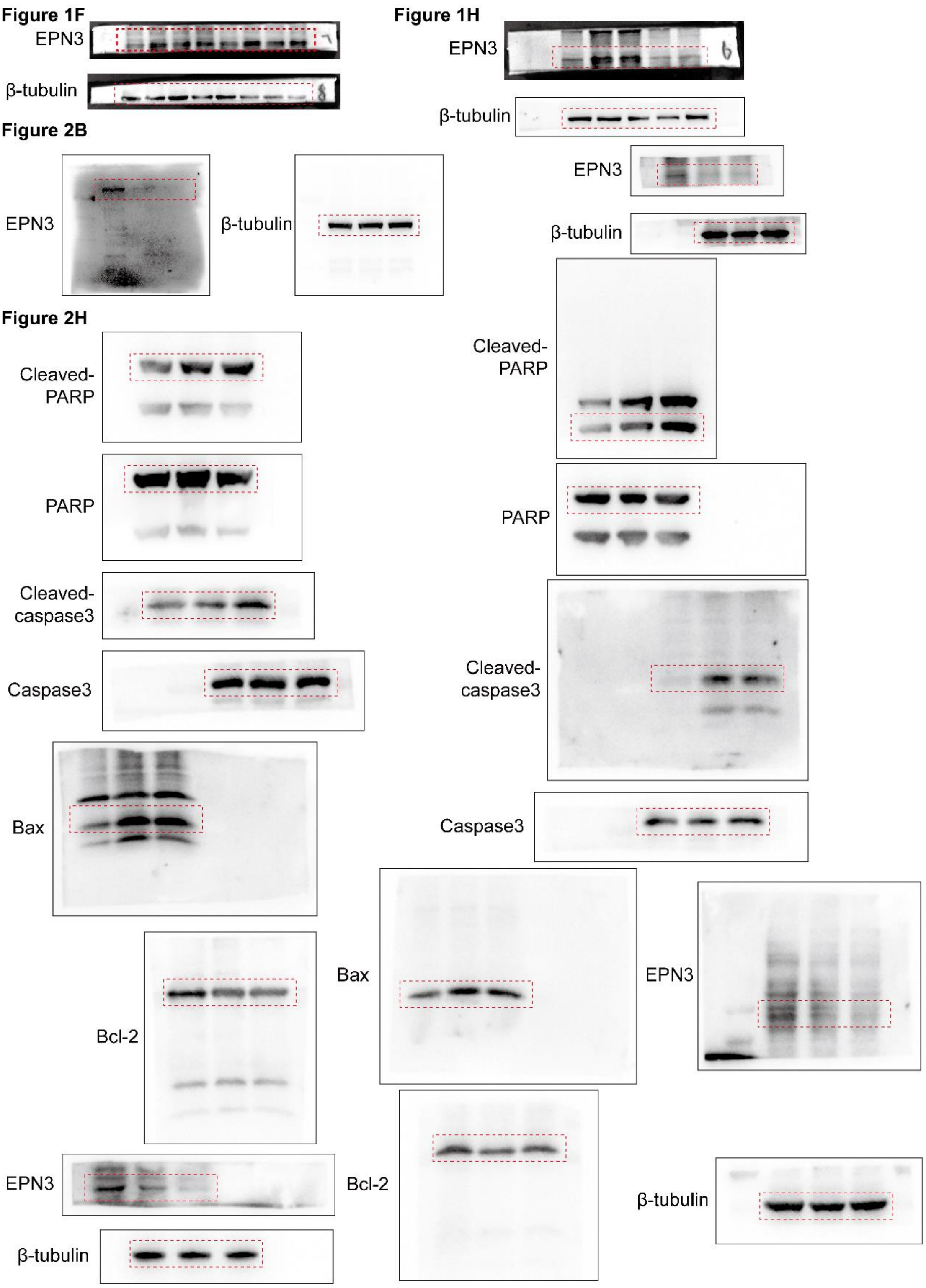

Figure 1F
EPN3
β-tubulin
Figure 1H
EPN3
β-tubulin
EPN3
β-tubulin
Figure 2B
EPN3
β-tubulin
Figure 2H
Cleaved-PARP
PARP
Cleaved-caspase3
Caspase3
Bax
Bcl-2
EPN3
β-tubulin
Cleaved-PARP
PARP
Cleaved-caspase3
Caspase3
Bax
EPN3
Bcl-2
β-tubulin

Figure 3E

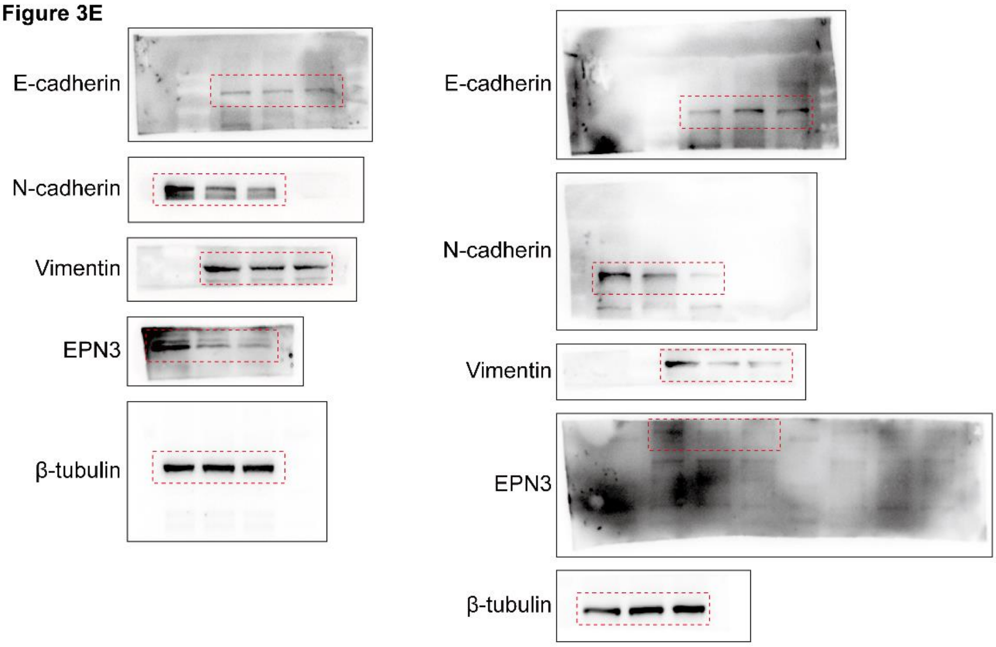

Figure 4D

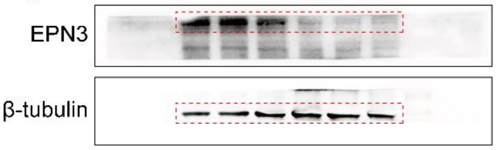

Figure 4I

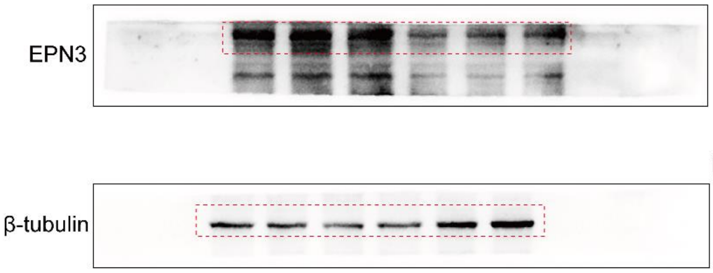

Figure 4J

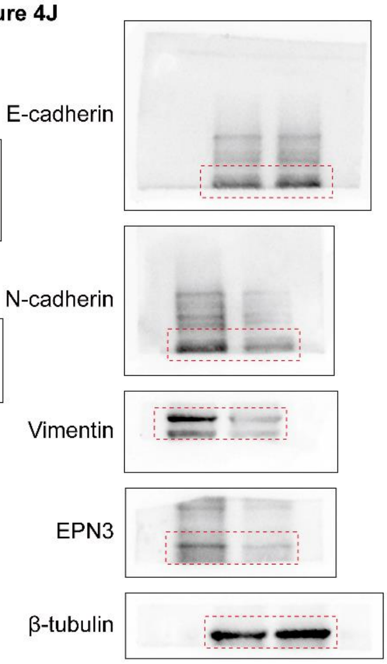

Figure 5C

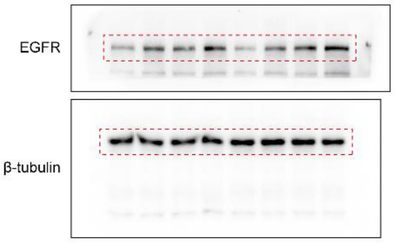

Figure 5D

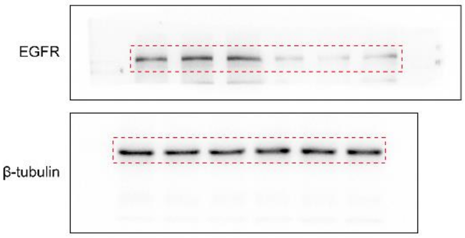

Figure 5F

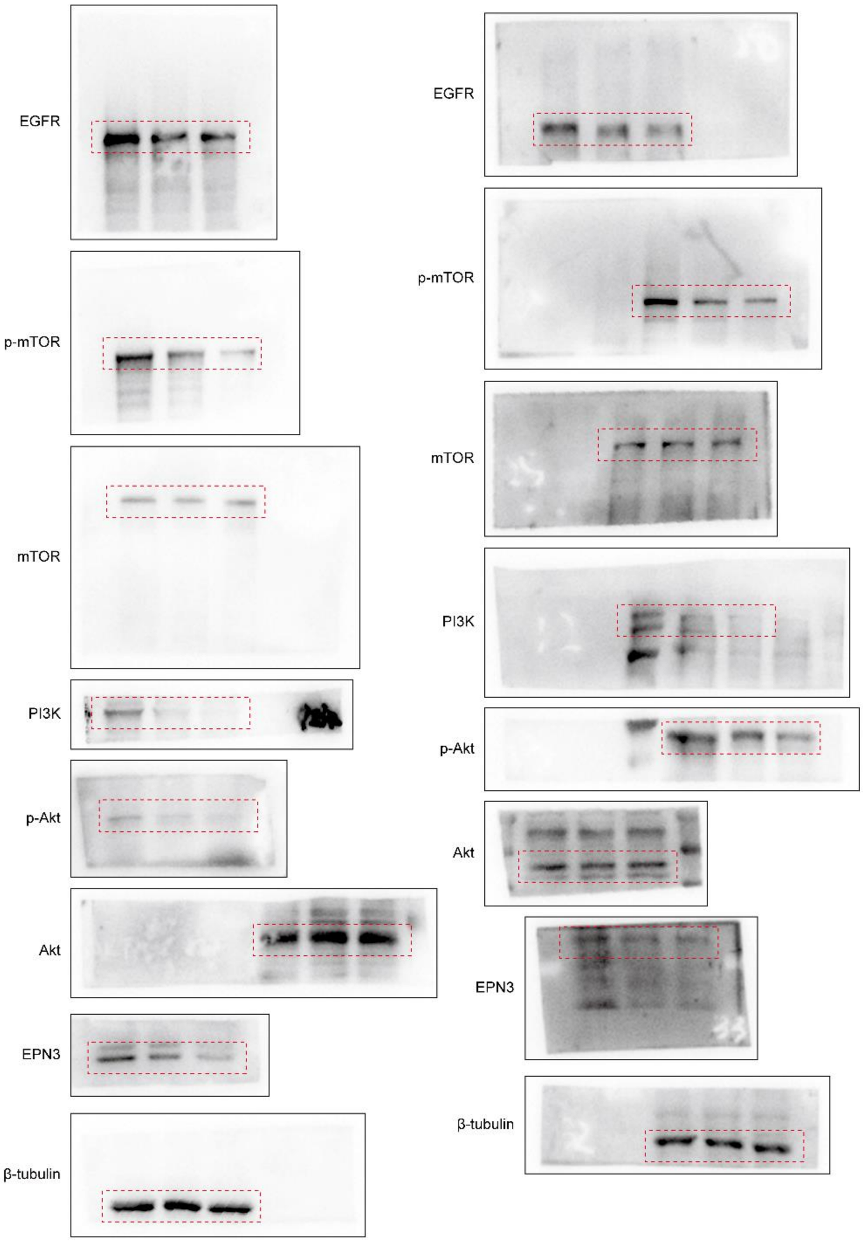

Figure 5F

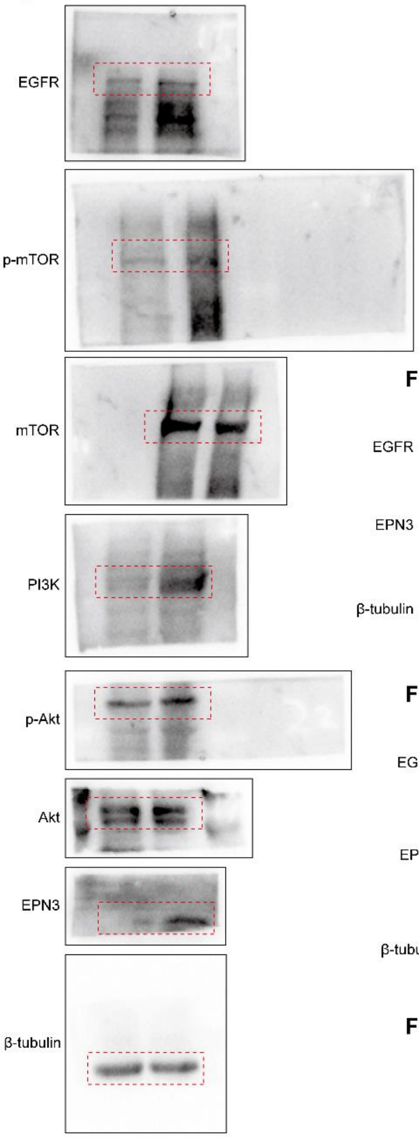

Figure 5G

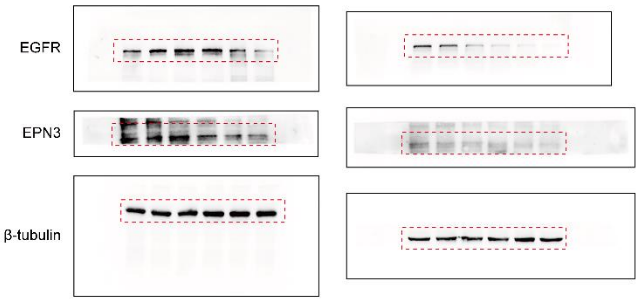

Figure 5H

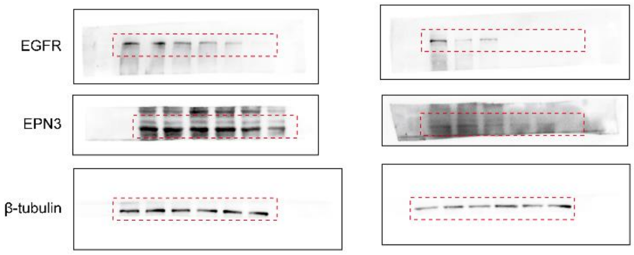

Figure 5I

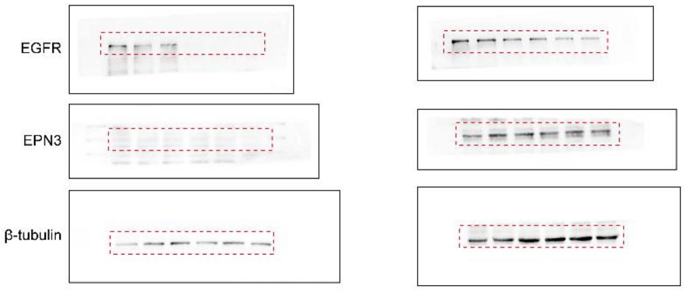

Figure 5J

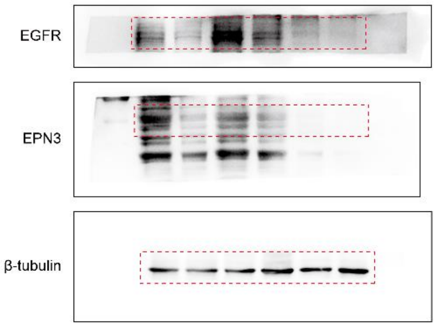

Figure 6B

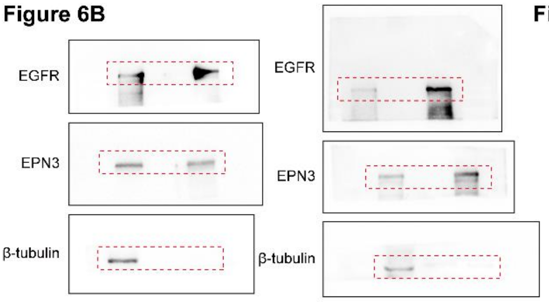

Figure 6C

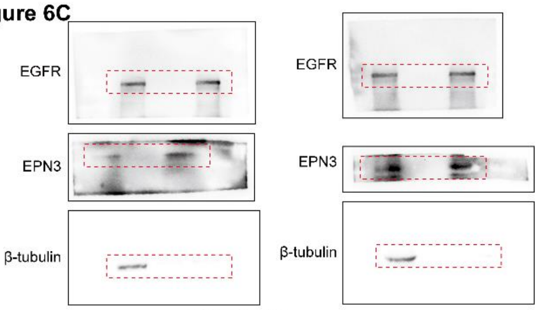

Figure 6D

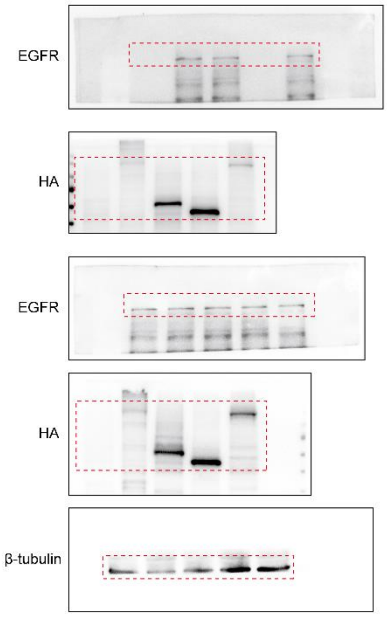

Figure 6E

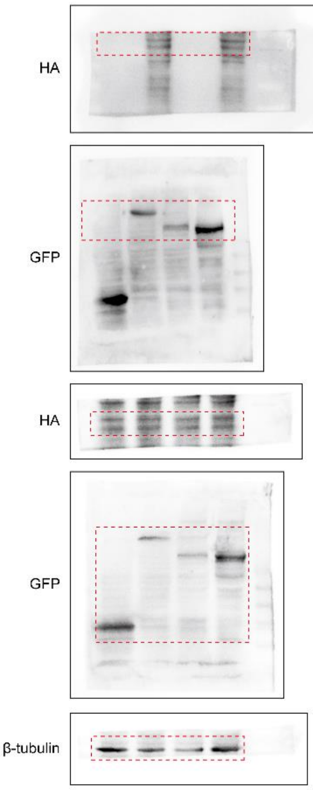

Figure 6F

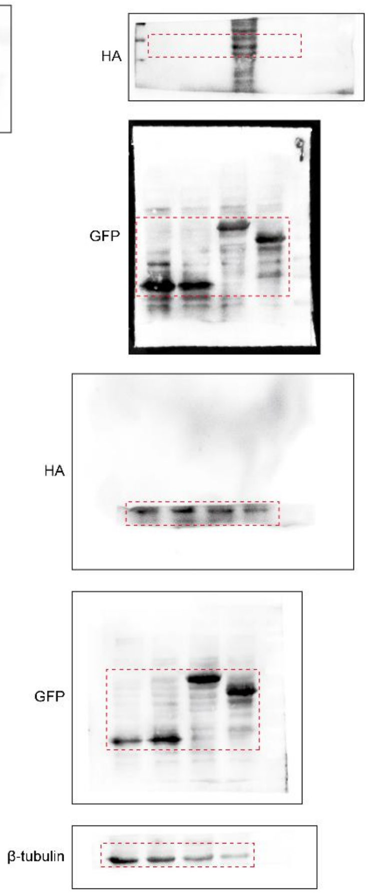

Supplement: Supplementary file 3 — Supplementary Material 3: Supplementary Figure 3 Exogenous EPN3 and EGFR were colocalized in NSCLC cells. [file 13578_2025_1358_MOESM3_ESM.pdf]
